# Sirtuin 4 activates autophagy and inhibits tumorigenesis by upregulating the p53 signaling pathway

**DOI:** 10.1038/s41418-022-01063-3

**Published:** 2022-10-08

**Authors:** Juan Li, Hanxiang Zhan, Yidan Ren, Maoxiao Feng, Qin Wang, Qinlian Jiao, Yuli Wang, Xiaoyan Liu, Shujun Zhang, Lutao Du, Yunshan Wang, Chuanxin Wang

**Affiliations:** 1 Department of Clinical Laboratory, The Second Hospital of Shandong University, 247 Beiyuan Street, Jinan, Shandong 250033 China; 2 Department of General Surgery, Qilu Hospital of Shandong University, 107 Wenhua Xi Road, Jinan, Shandong 250012 China; 3 Department of Anesthesiology, Qilu Hospital of Shandong University, 107 Wenhua Xi Road, Jinan, Shandong 250012 China; 4 Shandong Institute of Medical Device and Pharmaceutical Packaging Inspection, 15166 Century Avenue, Jinan, Shandong 250101 China

**Keywords:** Tumour-suppressor proteins, Autophagy, Cancer models

## Abstract

The role of autophagy in cancer is context-dependent. In the present study, we aimed to investigate the regulator and underlying mechanism of autophagy. We found that a sirtuin (SIRT) family member, SIRT4, was significantly associated autophagy pathway in pancreatic ductal adenocarcinoma (PDAC). Specifically, in vitro cell culture experiments and in vivo transgenic and xenografted animal models revealed that SIRT4 could inhibit tumor growth and promote autophagy in PDAC. In terms of the mechanism, we demonstrated that SIRT4 activated the phosphorylation of p53 protein by suppressing glutamine metabolism, which was crucial in SIRT4-induced autophagy. AMPKα was implicated in the regulation of autophagy and phosphorylation of p53 mediated by SIRT4, contributing to the suppression of pancreatic tumorigenesis. Notably, the clinical significance of the SIRT4/AMPKα/p53/autophagy axis was demonstrated in human PDAC specimens. Collectively, these findings suggested that SIRT4-induced autophagy further inhibited tumorigenesis and progression of PDAC, highlighting the potential of SIRT4 as a therapeutic target for cancer.

## Introduction

Autophagy is a highly conserved metabolic mechanism throughout evolution [1]. Both endogenous and exogenous stimuli can induce autophagy to maintain the stability of the internal environment [2]. In recent years, numerous studies have shown that autophagy dysfunction is implicated in the occurrence and development of tumors [3]. Some studies have shown that autophagy can prevent early cancer development and promote the progression of advanced cancer [4]. Therefore, understanding the biological role of autophagy helps explore the tumorigenesis of cancers.

p53 plays a complex and important regulatory role in cancer development. The reduction or loss of p53 function may lead to the development of cancer [5, 6]. p53 protein level is greatly elevated and activated in response to adverse signaling stimuli (such as DNA damage) [7]. The phosphorylation of p53 contributes to not only the stabilization but also the activation of the p53 [8]. Activated p53 is involved in the regulation of various physiological activities, such as cell cycle blockade, apoptosis, and metabolism [9, 10]. It has been shown that p53 plays a dual regulatory role in the regulation of autophagy. Nuclear p53 can either mediate autophagy promotion or repression by transcriptional regulation [11, 12], whereas cytoplasmic p53 inhibits autophagy in a transcription-independent manner [13]. It is very important to understand the mechanism of p53 on autophagy in tumors.

AMP-activated protein kinase (AMPK) maintains cellular energy homeostasis by regulating the metabolism of glucose, lipid, and protein [14]. The metabolic stress and energy imbalance increase the AMP/ATP ratio, which activates AMPK by phosphorylating α-subunit [15]. Subsequently, AMPK phosphorylates downstream target proteins to maintain energy homeostasis [16, 17]. AMPK has been linked to the manipulation of tumorigenesis by regulating autophagy, cell cycle, and apoptosis [18]. Accumulating evidence shows that AMPK plays a dual role in cancer [19]. Hence, an in-depth study of the mechanism of AMPK is required in tumor progression.

SIRT4 is an important member of the SIRT family, a class of highly conserved nicotinamide adenine dinucleotide (NAD+)-dependent deacetylases, and numerous studies have demonstrated the implication of SIRTs in tumor metastasis [20–22]. In our previous studies, we have found that SIRT4 can inhibit the metabolism of glutamine and activate AMPK, ultimately suppressing hepatocarcinogenesis [23]. However, its role in pancreatic ductal adenocarcinoma (PDAC) has not yet been studied. Recent studies have demonstrated that SIRT4 knockout (KO) mice have increased insulin secretion and ultimately glucose tolerance and insulin resistance [24, 25], all of which are high-risk factors for PDAC, suggesting that SIRT4 is closely associated with PDAC. Therefore, it is important to elucidate the molecular mechanism of SIRT4 in PDAC progression.

In the present study, we determined that SIRT4 was down-regulated in PDAC, serving as a tumor suppressor. Additionally, SIRT4 could regulate the expressions of autophagy-related genes (ARGs) in PDAC cells. Integration of multi-omics approaches identified AMPK-p53 as the core downstream targets associated with SIRT4-mediated glutamine metabolism in PDAC. Further in vivo and in vitro experiments confirmed that SIRT4 inhibited glutamine metabolism to activate AMPK, which promoted the phosphorylation of p53 and finally led to autophagy to suppress the development of PDAC.

## Results

### The autophagy program is linked to SIRT4 in cancers

**Fig. 1:**
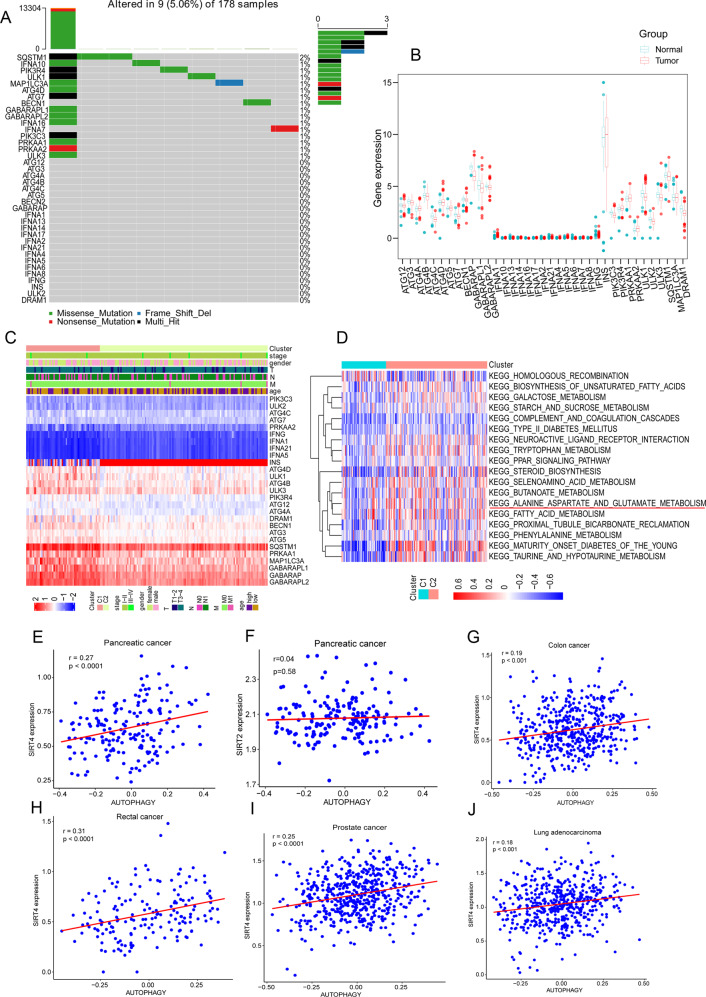
Genetic and transcriptional alterations of ARGs in PDAC. **A** Mutation frequencies of 38 ARGs in 178 PDAC patients from the TCGA cohort. **B** Expression distributions of 38 ARGs between normal and PDAC tissues. **C** Expressions of ARGs and clinicopathologic features between clusters 1 and 2. **D** GSVA of biological pathways between clusters 1 and 2, in which red and blue represent activated and inhibited pathways, respectively. **E**, **G**–**J**. Scatter plots showing correlation of transcript expression between SIRT4 and autophagy pathway in different tumors. **F** Scatter plots showing correlation of transcript expression between SIRT2 and autophagy pathway in PDAC.

We selected 38 ARGs and firstly explored ARG mutations in PDAC from The Cancer Genome Atlas (TCGA) simple nucleotide variation (SNV) data. Summary analysis of the incidence of somatic mutations in the 38 ARGs showed a relatively low mutation frequency in the PDAC cohort (Fig. 1A). Of the 178 PDAC samples, only nine (5.06%) had mutations in the ARGs (Fig. 1A), indicating that the mutations of ARGs had little relationship with PDAC. We further compared the expressions of ARGs at the mRNA level in PDAC and normal tissues and found that the expressions of most ARGs, such as ATG4C, GABARAP, DRAM1, and ULK1-3, were down-regulated in PDAC samples compared with normal samples (Fig. 1B). This finding indicated that autophagy might exert a suppressive effect on the tumorigenesis and development of PDAC. We used an unsupervised clustering algorithm to categorize the PDAC patients based on the expression profiles of the 38 ARGs [26]. We obtained two clusters (C1 = 54 and C2 = 123), and clinical data were indicated by the annotation bars above the heatmap (Fig. 1C). To investigate the pathways associated with autophagy, gene set variation analysis (GSVA) was applied to the two clusters. The results showed that cluster 2 was significantly enriched in metabolism-activated pathways such as alanine aspartate and glutamate metabolism, galactose metabolism, and fatty acid metabolism (Fig. 1D), indicating a close association between autophagy and metabolism. This observation was consistent with the diverse metabolic fuel sources that can be produced by autophagy [27, 28].

It has been reported that SIRT family members (SIRT1-SIRT7) are associated with glutamine metabolism [29, 30], while there are relatively few reports about the relation between the SIRT family and autophagy in PDAC. We examined the correlation between the SIRT family and the autophagy pathway using TCGA RNA-seq data. The results revealed that the expression of SIRT4, but not the other SIRTs, had the most significant positive association with the autophagy program in PDAC (Fig. 1E, F and Fig. S1A–E). We also detected a positive correlation between SIRT4 and autophagy pathways in other adenocarcinomas, including colon cancer, rectal cancer, prostate cancer, and lung adenocarcinoma (Fig. 1G–J). Taken together, these data suggested that the autophagy program was associated with PDAC progression, and SIRT4 was a candidate driver of the autophagy aberration.

### SIRT4 inhibits tumor growth and promotes autophagy in PDAC

**Fig. 2:**
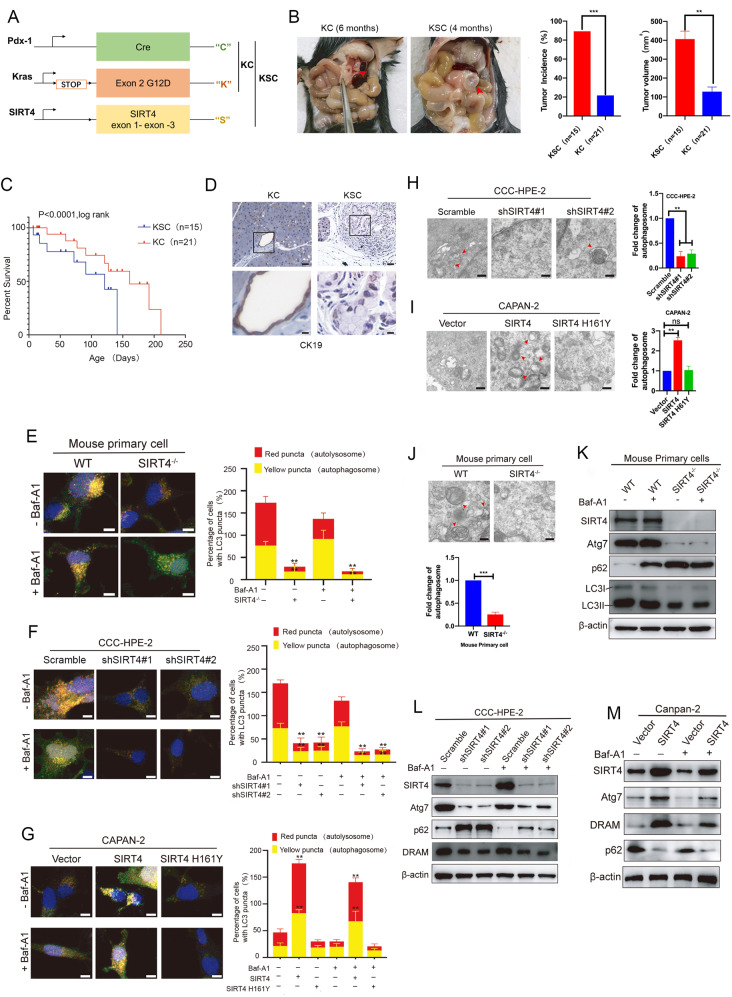
SIRT4 inhibits tumor growth and promotes autophagy in PDAC. **A** Construction of a mouse model that produced spontaneous pancreatic tumor with pancreas-specific depletion of SIRT4. **B** Representative images of pancreatic tumor formation in 6-month KC mice and 4-month KSC mice, and tumor incidence and tumor volume in KC (*n* = 21) and KSC (*n* = 15) mice. **C** Kaplan–Meier analysis of overall survival of KC (*n* = 21) and KSC (*n* = 15) mice. **D** Representative images of IHC staining showing the expression level of CK19 in pancreatic tissues of KC mice and KSC mice. Detection of the autophagic flux with the mRFP-GFP-LC3 reporter in mouse primary cells (**E**), CCE-HPE-2 (**F**), and Capan-2 (**G**). The indicated cells were separately transfected with an mRFP-GFP-LC3 reporter with or w/o 10 nM Baf-A1 treatment to inhibit the fusion of autophagosomes and lysosomes. Confocal microscopy images merged with GFP, RFP, and DAPI (blue) fluorescence of representative cells were acquired (left panel), and the percentages of cells showing accumulation of yellow or red puncta were quantified (right panel). SEM was applied to detect autophagy in CCE-HPE-2 (**H**), Capan-2 (**I**), and primary mouse cells (**J**). Red arrows indicate autophagosomes or autolysosomes. Western blotting analysis was used to detect the expressions of ARGs in mouse primary cells (**K**), CCE-HPE-2 (**L**), and Capan-2 (**M**) with or w/o Baf-A1 treatment. GraphPad software was used for independence Sample *t*-test, (*P < 0.05; **P < 0.01; ***P < 0.001).

It is well known that SIRT4 is a tumor suppressor gene, and its expression is down-regulated in cancer [31, 32]. The Western blotting analysis and immunohistochemical (IHC) staining showed that the expression of SIRT4 was lower in the PDAC tissue compared with the normal pancreas tissue (Fig. S2A, B). We also detected the expression of SIRT4 using the GEMMs of PDAC. SIRT4 was down-regulated in KPC and KC tissues compared with the wild-type (WT) pancreas tissue (Fig. S2C, D). Furthermore, a lower level of SIRT4 was observed in PDAC cell lines, SW1990, Capan-2, Hs766T, HPAC, and Panc-1 compared with normal pancreatic cell lines HPDE6-C7 and CCC-HPE-2 (Fig. S2E). In summary, SIRT4 was down-regulated in PDAC tissues, GEMMs, and cell lines. In vitro experiments showed that overexpression of SIRT4 significantly inhibited the proliferation and clonogenic ability (Fig. S2F, G), whereas SIRT4 deficiency led to enhanced proliferation and clonogenic ability (Fig. S2F, G). To further clarify the role of SIRT4 in PDAC development, a group of KRAS^G12D^; PDX1-Cre (KC) mice were crossed with SIRT4 systemic knockout mice to establish a SIRT4-deficient mouse model of spontaneous pancreatic tumorigenesis (KSC) (Fig. 2A). The results showed that SIRT4 depletion significantly promoted pancreatic tumorigenesis in KSC mice compared with KC mice (Fig. 2B), and a significantly shorter survival time was observed in SIRT4-deficient mice compared with KC mice (Fig. 2C). The SC (Pdx1-Cre; SIRT4^−/−^) mice had no developing solid tumor in pancreas and other tissues, and no dead during the period of our observation. We also observed that SIRT4 depletion induced the expression of cytokeratin 19 (CK19) which has been identified as a marker of ductal epithelial cells in PDAC [33] (Fig. 2D). Totally, these results suggested that the loss of SIRT4 promoted the development of PDAC.

In order to clarify the correlation between SIRT4 and the autophagy program, Autophagic flux was measured in the mouse primary cells from WT and SIRT4^-/-^ mice, which were transfected with an mRFP-GFP-LC3 reporter. As expected, scanning electron microscopy (SEM) showed that SIRT4 depletion suppressed autophagic flux (Fig. 2E) and decreased the number of autophagosomes and/or autophagolysosomes compared with the WT mice (Fig. 2J). In addition, we examined the occurrence of autophagy in CCC-HPE-2 and HPDE6-C7 cells with SIRT4 knockdown and Capan-2 and Hs766T cells with SIRT4 overexpression. We found that downregulation of SIRT4 inhibited autophagic flux and the production of autophagosomes and/or autophagolysosomes (Fig. 2F, H and Fig. S2H, I), while upregulation of SIRT4 promoted the production of autophagosomes (Figs. 2G, I and Fig. S2J, K). Western blotting analysis showed that the depletion of SIRT4 significantly reduced the expressions of Atg7, LC3I, LC3II, and DRAM and increased the expression of p62 (Fig. 2K, L and Fig. S2L). In contrast, the overexpression of SIRT4 significantly increased the expressions of Atg7 and DRAM and reduced the expression of p62 on protein level (Fig. 2M and Fig. S2M). These results suggested that SIRT4 induced endogenous autophagy in PDAC cells.

### SIRT4 affects the phosphorylation of p53 protein by regulating glutamine metabolism

**Fig. 3:**
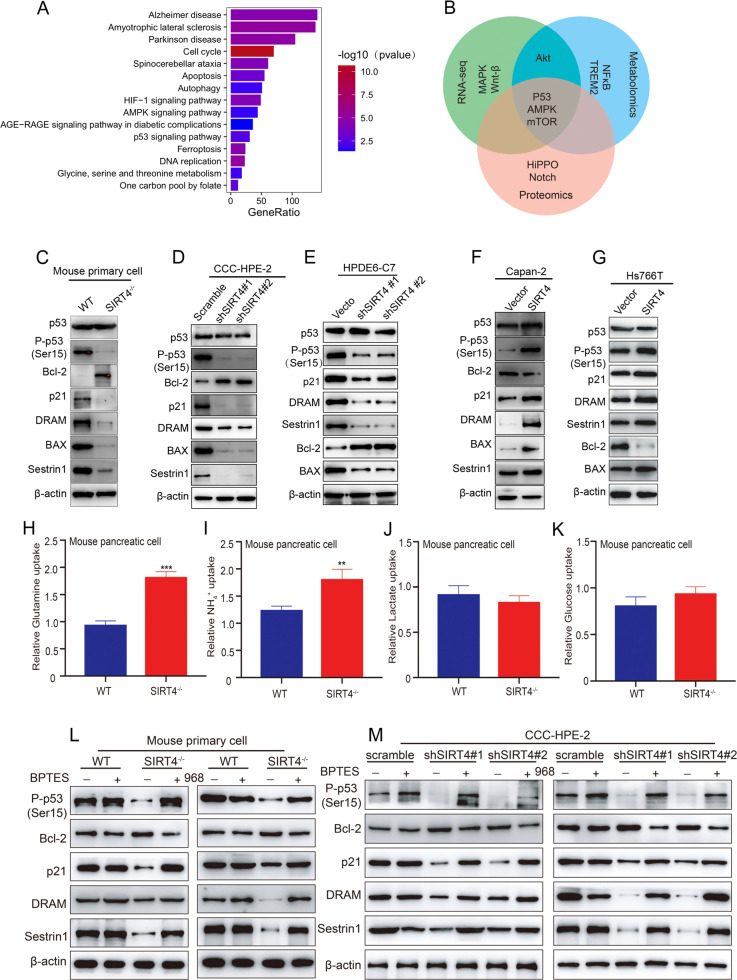
p53 phosphorylation mediates SIRT4-induced autophagy in PDAC. **A** KEGG analysis of the differentially expressed genes in Capan-2 cell with or without SIRT4 overexpression. **B** The intersections of pathways from metabolomics and RNA-seq with SIRT4-overexpressed Capan-2 cells, and proteomics in KSC mice. Western blotting analysis was used to detect the phosphorylation level of p53 protein and the expressions of downstream target genes in mouse primary cells (**C**), CCE-HPE-2 (**D**), and HPDE6-C7 cells (**E**). Western blotting analysis was used to detect the phosphorylation level of p53 protein and the expressions of downstream target genes in Capan-2 (**F**) and Hs776T cells (**G**). Detection of glutamine uptake (**H**), NH4^+^ production (**I**), lactate production (**J**), and glucose uptake (**K**) in primary pancreatic cells of WT mice and SIRT4^−/−^ mice. In the absence or presence of glutamine metabolic inhibitors, western blotting analysis was used to detect the phosphorylation level of p53 protein and the expressions of downstream target genes in mouse primary cells (**L**) and CCE-HPE-2 cells (**M**). GraphPad software was used for independence Sample *t*-test, (*P < 0.05; **P < 0.01; ***P < 0.001).

To further clarify how SIRT4 induced autophagy in PDAC cells, we performed RNA sequencing and analyzed the gene expression profile in the Capan-2 cells with or without SIRT4 overexpression. KEGG pathway enrichment analysis showed that SIRT4 was involved in regulating the AMPK signaling pathway, p53 signaling pathway, and cell cycle (Fig. 3A). Combined with the proteomics and metabolomics analysis, we found that SIRT4 was involved in regulating the activation of the p53, AMPK, and mTOR pathways (Fig. 3B). We next experimentally explored whether SIRT4 was involved in regulating the p53 signaling pathway. We found that depletion of SIRT4 reduced the p53 phosphorylation but had no effect on the total p53. The expressed changes of p53 target genes were also observed with SIRT4 knockdown, including reduced p21, DRAM, BAX, and Sestrin1 and increased Bcl-2 protein (Fig. 3C–E). Moreover, contradictory trends were found when SIRT4 was overexpressed (Fig. 3F, G). These results were further confirmed in mouse pancreatic tissues by IHC staining (Fig. S3A). These data demonstrated that SIRT4 promoted the phosphorylation of p53 protein.

Previous studies have shown that SIRT4 can participate in regulating glutamine metabolism [31, 32]. In the present study, we found that SIRT4-deleted mouse pancreatic primary cells exhibited significantly increased glutamine uptake and NH4^+^ production (Fig. 3H, I), while the glucose uptake and lactate production remained unaffected (Fig. 3J, K), suggesting that the loss of SIRT4 increased the ability of cells to use glutamine. Similar observations were also found in human PDAC cells with stable SIRT4 overexpression or knockdown (Fig. S3B–E). To further investigate whether SIRT4 regulated the phosphorylation of p53 protein via inhibiting glutamine metabolism, we applied glutaminase inhibitors BPTES and 968 to treat primary mouse cells and CCC-HPE-2 cells. The results confirmed that BPTES and 968 increased the phosphorylation of p53, and thereby affected the expressions of p53 downstream target genes (Fig. 3L, M). These results illustrated that the phosphorylation of p53 protein activated by SIRT4 was associated with the inhibition of glutamine metabolism.

### p53 phosphorylation mediates SIRT4-induced autophagy in PDAC

**Fig. 4:**
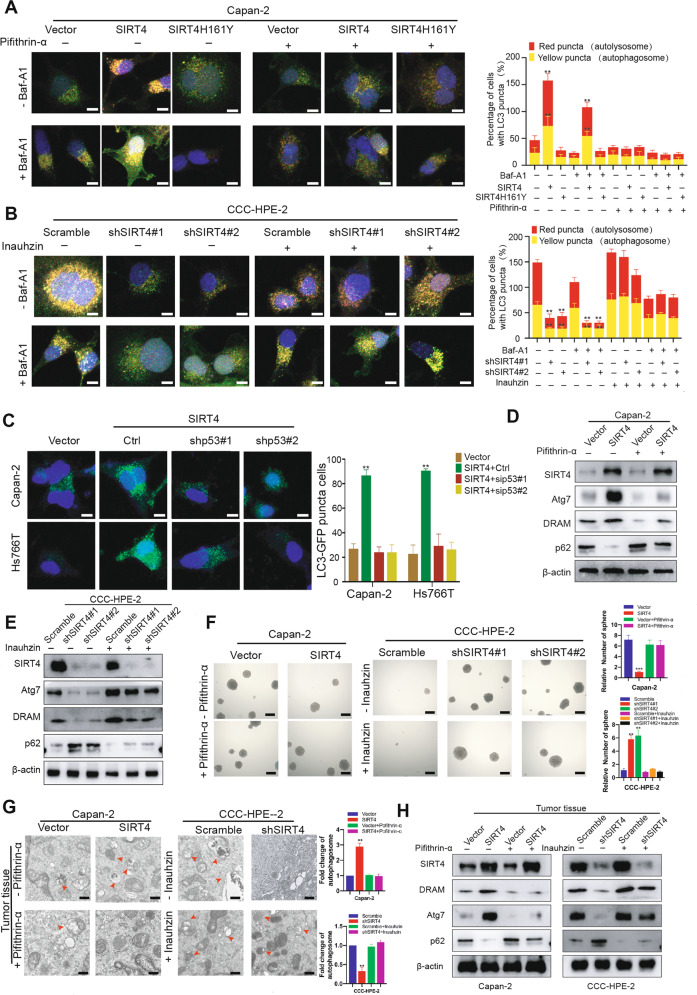
p53 phosphorylation mediates SIRT4-induced autophagy in PDAC. **A** Representative images (left) and its quantification (right panel) of the autophagic flux detection with the mRFP-GFP-LC3 reporter in Capan-2 cells co-transfected with control, SIRT4, or SIRT4H161Y vectors, respectively, in the absence or presence of pifithrin-α, the inhibitor of p53 signaling pathway. **B** Representative images (left) and its quantification (right panel) of the autophagic flux detection with the mRFP-GFP-LC3 reporter in CCC-HPE-2 cells co-transfected with scramble or sh-SIRT4 vectors, respectively, in the absence or presence of Inauhzin, the activator of p53 signaling pathway. **C** In PDAC cell lines overexpressing SIRT4, the expression of p53 was transiently depleted, and LC3-GFP puncta in cells were observed. **D** After p53 signaling pathway inhibitors were used to treat PDAC cells overexpressing SIRT4 and its control cell line, the expressions of ARGs were detected by western blotting analysis. **E** SIRT4-depleted PDAC cell lines and control cell lines were treated with a p53 signaling pathway activator, the expressions of ARGs were detected by western blotting analysis. **F** Soft Agar technology was used to observe the effect of the p53 signaling pathway on the function of SIRT4. **G** SEM was applied for the detection of autophagy in tumor tissues of nude mice subcutaneously injected with Capan-2 and CCC-HPE-2 cells mentioned above. **H** Western blotting analysis was used to detect the expressions of ARGs in tumor tissues of nude mice subcutaneously injected with Capan-2 and CCC-HPE-2 cells mentioned above. GraphPad software was used for independence Sample t-test, (*P < 0.05; **P < 0.01; ***P < 0.001).

Given the important role of p53 in cell autophagy [34, 35] and the impact of SIRT4 on p53 phosphorylation (Fig. 3), we speculated that SIRT4 might promote autophagy via activating p53. To verify such hypothesis, p53 signaling pathway inhibitor (Pifithrin-α) and activator (Inauhzin) were used to treat SIRT4-overexpressing cells (Capan-2 and Hs766T) and SIRT4-deficient cells (CCC-HPE-2 and HPDE6-C7), respectively. We used an mRFP-GFP-LC3 reporter to reflect autophagic flux and detected the effect of p53 activation on SIRT4-mediated autophagy. The results indicated that the autophagic flux was increased upon SIRT4 overexpression, while Pifithrin-α reversed this effect (Fig. 4A and Fig. S4A). On the contrary, Inauhzin could reverse the autophagy inhibition caused by SIRT4 deficiency (Fig. 4B and Fig. S4B). This result was further confirmed by GFP-LC3 expression, which was inhibited in SIRT4-overexpressing cells with p53 knockdown and upregulated in SIRT4-depleted cells treated by p53 activator Inauhzin (Fig. 4C and Fig. S4C). Similar effects on the regulation of p53-mediated autophagy by SIRT4 were observed by Western blotting analysis (Fig. 4D, E). These data indicated that SIRT4 promoted the autophagy pathway by enhancing p53 phosphorylation. In addition, we found that changes in the p53 signaling pathway reversed the changes in the spherogenic ability caused by SIRT4 overexpression or depletion (Fig. 4F). Furthermore, cells treated with Pifithrin-α and Inauhzin were subcutaneously injected into nude mice to observe the tumorigenic ability of the cells in vivo. The results showed that the tumor growth was suppressed when SIRT4 was overexpressed, while Pifithrin-α reversed this effect, and the Inauhzin could restore the tumor growth caused by SIRT4 depletion (Fig. S4D–G). We also used transmission electron microscopy and Western blotting analysis to assess the autophagy in mouse tumor tissues, confirming that p53 played an important role in SIRT4-induced cell autophagy (Fig. 4G, H). Collectively, these data suggested that SIRT4 induced autophagy in a p53-dependent manner in PDAC cells.

### AMPK mediates SIRT4-regulated autophagy and regulates p53 phosphorylation in PDAC

**Fig. 5:**
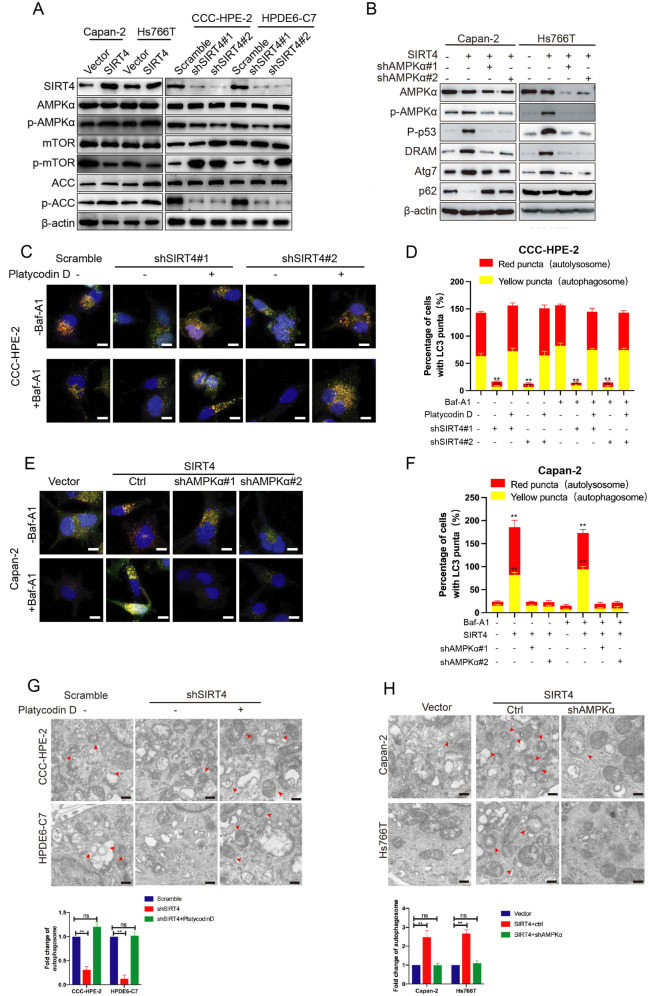
AMPK mediates SIRT4-regulated autophagy and regulates p53 phosphorylation in PDAC. **A** Western blotting analysis was used to detect the expressions of AMPK and its downstream target proteins in PDAC cells when SIRT4 was overexpressed or depleted. **B** Western blotting analysis was used to detect whether the decrease of AMPK expression in PDAC cells could affect the phosphorylation of p53 protein and the expressions of ARGs in Capan-2 and Hs766T cells overexpressing SIRT4. **C**, **D** Representative images (left) and its quantification (right panel) of the autophagic flux detection with the mRFP-GFP-LC3 reporter in CCC-HPE-2 cells co-transfected with scramble or sh-SIRT4 vectors, respectively, in the absence or presence of platycodin D, an AMPK activator. The indicated cells were treated with or without 10 nM Baf-A1 inhibited autophagosome-lysosome fusion. **E**, **F** After AMPK was depleted in Capan-2 cell lines overexpressing SIRT4, the mRFP-GFP-LC3 dual fluorescence labeling method was applied to detect the occurrence of autophagy. The indicated cells were treated with or without 10 nM Baf-A1 inhibited autophagosome-lysosome fusion. **G** SEM was applied to detect autophagy in CCC-HPE-2 and HPDE6-C7 cells. **H** SEM was applied to detect autophagy in Capan-2 and Hs766T cells. GraphPad software was used for the independence sample *t*-test (*P < 0.05; **P < 0.01).

Our previous study has found that SIRT4 can activate AMPK by regulating glutamine metabolism and ultimately inhibit hepatocarcinogenesis [23]. We aimed to determine whether SIRT4 played the same role in PDAC. We found that the ectopic expression of SIRT4 significantly activated AMPKα and ACC, reducing the activation of mTOR (Fig. 5A). Consistently, depletion of SIRT4 significantly reduced the phosphorylation of AMPKα and ACC and increased the phosphorylation of mTOR (Fig. 5A). We then detected the effect of AMPKα on p53 phosphorylation and expressions of ARGs induced by SIRT4. AMPKα depletion impaired the P-p53, DRAM, and Atg7 proteins enhanced by SIRT4 overexpression and increased the expression of p62 reduced by SIRT4 overexpression (Fig. 5B). This result was further confirmed by qRT-PCR (Fig. S5A–D). The AMPK activator platycodin D significantly increased the autophagic flux in pancreatic epithelial cells after SIRT4 knockdown (Fig. 5C, D, S6A, C). Moreover, depletion of AMPKα in SIRT4-overexpressing PDAC cells could significantly inhibit SIRT4-induced autophagy (Fig. 5E, F, S6B, D). The above results were further confirmed by the autophagosome formation in the cells (Fig. 5G, H). In conclusion, our study indicated that AMPK played an important role in p53 phosphorylation and SIRT4-regulated autophagy in PDAC cells.

### AMPKα plays a key role in SIRT4-suppressed PDAC development

**Fig. 6:**
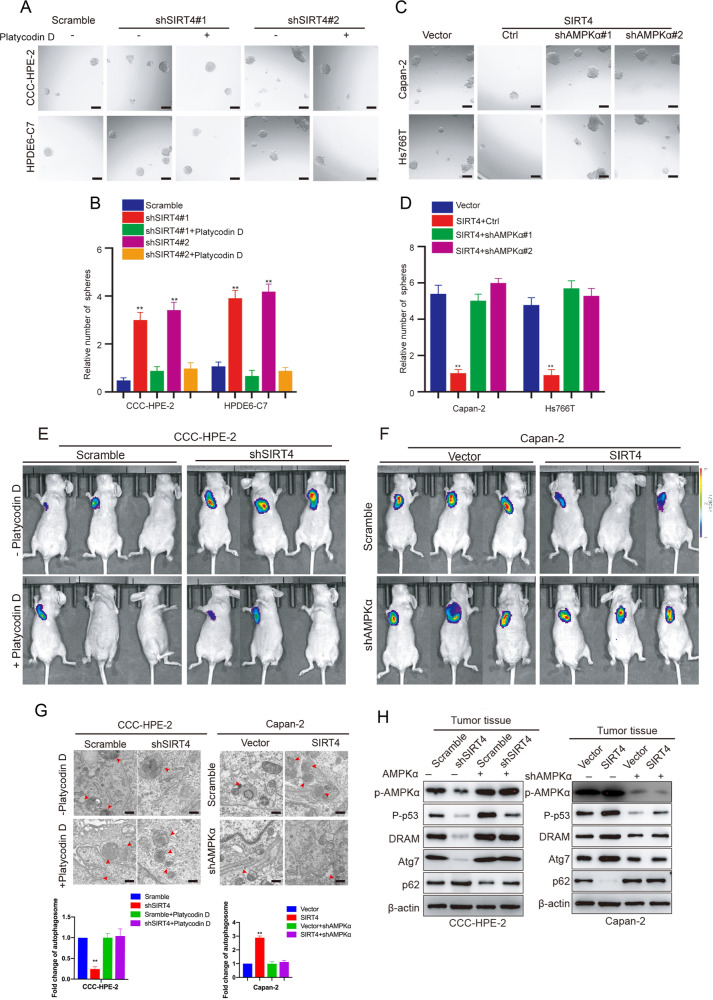
AMPKα plays a key role in the suppression of pancreatic development by SIRT4. **A**, **B** Representative images and its quantification of sphere formation assay to detect the tumorigenic ability of CCC-HPE-2 and HPDE6-C7 cells transfected with scramble or sh-SIRT4 vectors, respectively, in the absence or presence of platycodin D, an AMPK activator. **C**, **D** Representative images and its quantification of sphere formation assay to detect the tumorigenic ability of Capan-2 and Hs766T cells bearing control or SIRT4 vectors, respectively, in response to AMPK depletion. Representative ventral view images of bioluminescence from nude mice subcutaneously injected with CCC-HPE-2 (**E**) and Capan-2 cells (**F**). **G** SEM was applied to detect autophagy in tumor tissues of nude mouse xenograft tumor models. **H** Western blotting analysis was used to detect the expressions of ARGs in tumor tissues of nude mouse xenograft tumor models. GraphPad software was used for the independence sample t-test (*P < 0.05; **P < 0.01).

To clarify the role of AMPKα in SIRT4-regulated PDAC, genetic overexpression and pharmaceutical activation of AMPK were carried out in SIRT4-deficient cells, and genetic ablation and pharmaceutical inhibition of AMPK were applied in SIRT4-overexpressing cells. The sphere formation assay showed that upregulation of AMPK in SIRT4-depleted cells could significantly inhibit the tumorigenic ability of PDAC cells (Fig. 6A, B, and Fig. S7A, B). Conversely, downregulation of AMPKα in SIRT4-overexpressing cells could promote the spherogenic ability of PDAC cells (Fig. 6C, D and Fig. S7C, D). The same results were observed in SIRT4-depleted mouse primary cells overexpressing AMPKα or treated with platycodin D (S7E, H). Using a xenograft tumor model in nude mice, we also observed that the changes in AMPK expression could reverse the effect of SIRT4 expression on the tumorigenic ability of PDAC cells (Fig. 6E, F). Further ultrastructural evidence of autophagy in mouse tumor tissues confirmed that the AMPKα was implicated in the induction of autophagy by SIRT4 in PDAC cells (Fig. 6G). Western blotting analysis showed that AMPKα was associated with SIRT4-promoted phosphorylation of p53 (Fig. 6H). In a word, activation of AMPKα was a key step for SIRT4 to regulate p53 phosphorylation and inhibit PDAC.

### The SIRT4/AMPKα/p53 axis is of great clinical significance in PDAC patients

**Fig. 7:**
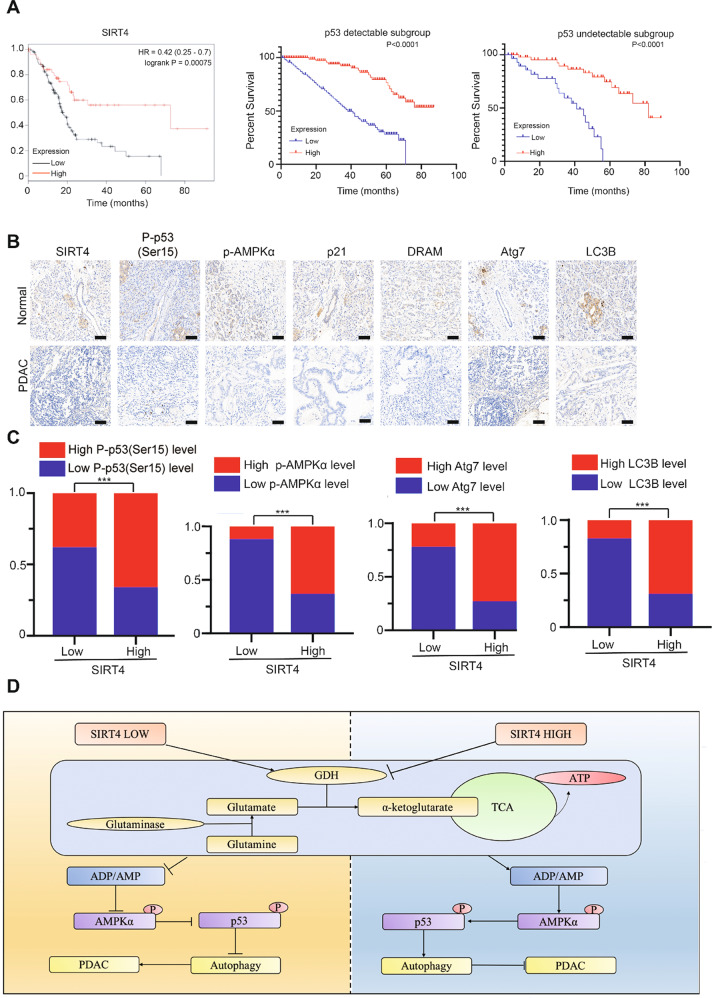
The SIRT4/AMPKα/p53 axis is of great clinical significance in PDAC patients. **A** Kaplan–Meier analysis of overall survival of PDAC patients with different expressions of SIRT4 and p53. **B** Representative images of IHC staining to detect the expressions of related proteins in clinical tissue samples of PDAC patients. **C** Statistical analysis of related protein expression levels and SIRT4 expression levels in clinical tissue samples of PDAC patients. **D** Schematic diagram of SIRT4-AMPK-p53 axis in regulating autophagy and PDAC development. GraphPad software was used for independence Sample *t*-test, (*P < 0.05; **P < 0.01; ***P < 0.001).

To clarify the clinical significance of the SIRT4/AMPKα/p53 axis in PDAC, we analyzed the association between SIRT4 or p53 and the survival of PDAC patients and found that the median survival time of PDAC patients with high SIRT4 expression was significantly prolonged, while the median survival time of patients with low SIRT4 expression was significantly decreased. Moreover, the prolonged prognostic significance of high SIRT4 expression was observed in both detectable p53 and undetectable p53 groups (Fig. 7A), indicating that p53 might be not the only target of SIRT4 and SIRT4 could function in cancer by other targets. The expressions of P-p53, AMPKα, and their downstream proteins, as well as ARGs, were detected in clinical tissue samples (Fig. 7B), and the correlation between their levels and SIRT4 expression was assessed (Fig. 7C). Taken together, these clinical data strongly suggested that downregulation of SIRT4 was associated with the decreased expressions of P-p53, p-AMPKα, and ARGs, which might be linked to the poor clinical outcomes of PDAC patients.

## Discussion

Autophagy is associated with various forms of human diseases, including cancers [2]. Dysregulated autophagy causes the accumulation of damaged organelles and misfolded proteins to initiate DNA damage, leading to tumorigenesis [36]. However, the precise mechanisms regulating autophagy in tumors are relatively less understood. Especially, autophagy is a dynamic and complex process and plays context-dependent roles in the inhibitory or promoting effects on cancer. Therefore, it is urgently necessary to understand the detailed mechanisms of autophagy in a specific type of cancer. In our current study, we found a key regulator, SIRT4, which could promote the autophagy program in a glutamine-AMPKα-p53-dependent manner, greatly contributing to inhibiting tumorigenesis in PDAC.

Some studies have shown that SIRT4 mainly exerts tumor-suppressive function and is correlated with a worse prognosis in multiple cancers [22, 37, 38]. Here, we reported its role as a tumor suppressor in PDAC, which could reduce cell proliferation and tumor growth. The low expression of SIRT4 also predicted a poorer prognosis in PDAC patients. Importantly, we revealed that the expression of SIRT4, but not the other SIRT members, had the most significant positive association with the autophagy program in PDAC. Moreover, we, for the first time, addressed its activation effect on autophagy in PDAC in vitro and in vivo. In line with our study, a recent study has found that the expression of SIRT4 promotes stress-triggered autophagic flux of mitochondria in HEK293 cells [39]. However, there is a discrepancy in the action modes of SIRT4 in PDAC versus cardiac injury or under normal physiological conditions. Previous studies have reported the anti-autophagic effect of SIRT4 in cardiac injury or under physiological conditions [40, 41]. Such opposite biological effects of SIRT4 on autophagy may be attributed to different context-dependent effects. It is, therefore, greatly interesting to further clarify which reasons lay a foundation for SIRT4 to differentially regulate cancer and other types of disease or physiology.

While interrogating how SIRT4 evokes autophagy in PDAC cells, we first found that SIRT4, which stimulated autophagy, activated the phosphorylation of p53 but had no effect on the total p53 in PDAC cells. On the other hand, silencing SIRT4 decreased autophagy, along with a reduction in phosphorylated p53. Blockade of p53 abolished the autophagy-promotion effect of SIRT4 overexpression. These results indicated that SIRT4 increased autophagy by phosphorylating p53 in PDAC cells. Previous studies have shown that p53 can regulate damaged cells to adapt to the stress response as a pro-autophagic factor in a transcription-dependent manner, while p53 knockdown or knockout by chemical inhibition or genetic deletion increases autophagy in both normal and transformed cells [42, 43]. Given the general complexity of p53 on autophagy, exploring its regulatory factors will provide important complementation to understand this regulatory process. Interestingly, we found that the prognostic value of SIRT4 in PDAC was not affected by the p53 state even though SIRT4 could regulate p53, indicating that p53 might be not the only target of SIRT4, and SIRT4 could exert its functions in cancer by other targets. However, such a hypothesis should be verified in further research.

AMPK plays a key role in the regulation of bioenergy metabolism [44]. The activity of AMPK is regulated by many factors, including glutamine metabolism. Our previous studies have unveiled that the inhibitory effect of SIRT4 on glutamine metabolism contributes to AMPK activation and suppression of hepatocarcinogenesis [23]. Consistently, we also confirmed that SIRT4 could inhibit glutamine metabolism to phosphorylated AMPK in PDAC cells. Moreover, in response to metabolic stress, AMPK has been reported to induce p53 via phosphorylating and inactivating MDMX [45]. In this study, we constructed a link between SIRT4 and phosphorylated p53 by AMPK. We illustrated that depletion of AMPKα upon SIRT4 overexpression in PDAC cells could reverse SIRT4-induced p53 phosphorylation and autophagy. Considering the importance of p53, AMPK, and autophagy in cancer cell biology, a comprehensive understanding of the interplay between these key molecules remains necessary for developing effective therapeutic strategies.

In conclusion, our research confirmed the tumor-suppressive function of SIRT4 in PDAC using a range of molecular, cell biology, animal, and clinical approaches. This study revealed a new mechanism of SIRT4-mediated autophagy in PDAC and preliminarily addressed the possibility of SIRT4 as a potential biomarker for prognostic prediction of PDAC patients.

## Materials and methods

### Patients and ethics statement

Samples were randomly gathered from PDAC patients who underwent curative resection at Qilu Hospital, Shandong University without preoperative radiation therapy or chemotherapy. The included PDAC specimens were all pathologically confirmed. All tissue specimens were collected immediately after tumor resection, snap-frozen in liquid nitrogen, and then stored at −80 °C. Study protocols complied with the Helsinki declaration and were approved by the Ethics Committee of Qilu Hospital of Shandong University. Written informed consents were obtained from all participants.

### Genetically engineered mouse model (GEMM)

The Kras^G12D/+^; Pdx1-Cre (KC) and Kras^G12D/+^; Trp53R^172H/+^; Pdx1-Cre (KPC) GEMM was used for treatment evaluation using the breeding strategy as follows. LSLKras^G12D^ (strain #01XJ6) mice were crossed with mutated LSL-p53^R172H^ mice (strain #01XM2) and pancreas-specific Cre mice (PDX1-Cre, strain #01XL5) to yield mice that possessed a conditional p53 mutation and endogenous levels of mutant Kras^G12D^ expressed by pancreatic tissue. Spontaneously developed PDAC was diagnosed by a palpable solid tumor in the left peritoneal cavity, which was verified by an MRI scan. When the mice presented with a hunched posture, hyperpnea or dyspnea, ascites, or lethargy, they were euthanized, and the survival data were recorded. SIRT4 KO mice were obtained from the Jackson Laboratory. All animals were randomly numbered. After data collection, genotypes were revealed, and animals were randomly allocated into each group. Investigators were blinded to group allocation during data collection and analysis. The sample size and inclusion criteria of each group were confirmed with adequate power based on the literature and our previous experience. None of the mice with the appropriate genotype were excluded from this study or used in any other experiments. Mice did not undergo prior treatment or procedures. All mice were fed a standard chow diet ad libitum and housed in a pathogen-free facility under standard conditions (controlled temperature, humidity, and 12-h light–dark cycle), and the animals were under the supervision of veterinarians with no more than five mice per cage. All mice were reared in the Animal Experiment Center of the Second Hospital of Shandong University. All experimental procedures were performed in accordance with the Guide for the Care and Use of Laboratory Animals published by the National Institutes of Health (NIH publication, 8th edition, 2011) and approved by the Institutional Animal Care and Use Committee of the Second Hospital of Shandong University review and approval.

### Cell cultures

Human PDAC cell lines, including Capan-2, Hs766T, and human normal pancreatic epithelial cells, CCC-HPE-2, HPDE6-C7, were purchased from ATCC. Mouse primary pancreatic epithelial cells were obtained from KPC, KSC, KC, and WT mice. PDAC cells and normal pancreatic epithelial cells were cultured using DMEM (Gibico) containing 10% fetal bovine serum (Gibico) and 20% fetal bovine serum (Gibico), respectively, in a cell incubator at 37 °C and with 5% CO_2_ saturated humidity. Mouse primary pancreatic epithelial cells were cultured using DMEM/F12 medium containing 20% fetal bovine serum in a cell incubator at 37 °C and 5% CO_2_ saturated humidity.

### Tissue specimens and immunohistochemistry

Adjacent normal pancreatic tissue specimens were collected at the standard distance (3 cm) from the edge of the excised tumor tissue of PDAC patients undergoing surgical pancreatectomy. All clinical PDAC specimens used in this study were histopathologically and clinically diagnosed. In order to use these clinical materials for research purposes, the patient’s consent and approval were obtained in advance from the Institutional Research Ethics Committee of the Qilu Hospital of Shandong University. The study conformed to all relevant ethical norms involving human participants. The specimens were fixed with formalin, paraffin-embedded sections, and the expression of SIRT4, CK19, P-p53, p-AMPK, p21, DRAM, Atg7, and LC3B were detected by immunofluorescence staining using specific antibodies. Two independent observers scored the proportion of positively stained tumor cells and the staining intensity. The cut-off values for high and low expression of the protein of interest are selected based on the measurement of heterogeneity using the log-rank test on overall survival.

### Lentivirus construction and infection

SIRT4 shRNAs, AMPKα shRNAs, p53 shRNAs, and nonsense control shRNAs were inserted into the plasmid vector GV248 and lentiviruses were constructed, which were purchased from Shanghai GeneChem Co., Ltd. Cells were infected with 8 mg/ml polybrene and selected with puromycin following the manufacturer’s instructions. Cells are then harvested for qRT-PCR or immunoblotting etc. The shRNA target sequences used in this study were: nonsense, 5′-TTCTCCGAACGTGTCACGT-3′; SIRT4, 5′-GAACCCTGACAAGGTTGATTT -3′; AMPKα, 5′-GTTGCCTACCATCTCATAATA-3′; P53, 5′-GCAUGAACCGGAGGCCCAU-3′.

### Immunoblotting

Cells or PDAC tissues were lysed using RIPA buffer containing protease inhibitor and phosphatase inhibitor. The expression of corresponding proteins was detected by sodium dodecyl sulfate-polyacrylamide gel electrophoresis (SDS-PAGE) gel electrophoresis, and Western Blot Imaging and band quantification were performed using ChemiDocTM MP Imaging System. β-actin was adopted as a loading control. All antibodies are listed in Supplementary Table 1. Full-length original western blots for these results are provided in Supplementary File 1.

### Quantitative Real-time (RT)-PCR (qRT-PCR)

Total RNA was isolated from cultured cells with TRIzol reagent (Invitrogen) as directed. According to the manufacturer’s instructions, PrimeScriptTM RT Reverse Transcription Kit (TaKaRa) was used to reverse the RNA (1 μg) to the cDNA (20 μl). The experiment was performed at least three times. Endogenous GAPDH was used as a standardized control. Quantitative analysis was performed by comparing CT values. The specific sequence primers were designed as follows: forward 5′-CAGCAAGTCCTCCTCTGGAC-3′ and reverse 5′-CCAGCCTACGAAGTTTCTCG-3′ for SIRT4; forward 5′-AAGCAACTCTGGATGGGATT-3′ and reverse 5′-GCAGCCACAGGACGAAAC-3′ for ATG5; forward 5′-GGTGTGGACAGATGATCTTTGC-3′ and reverse 5′-CCAACTCCCATTTGCGCTATC-3′ for ATG4; forward 5′-CAGTCCGTTGAAGTCCTC-3′ and reverse 5′-TCAGTGTCCTAGCCACATTAC-3′ for ATG7; forward 5′‐AGGCCCACGAGAACGAGT‐3′ and reverse 5′‐GGTGGAATTTTTGCCAATGT‐3′ for ATG16; forward 5ʹ-TGTCCGACTTATTCGAGAGCAG-3ʹ and reverse 5ʹ-TCACTCATGTTGACATGGTCAGG-3ʹ for LC3B; forward 5′-AGGTCGGTGTGAACGGATTTG-3′ and reverse 5′-GGGGTCGTTGATGGCAACA-3′ for GAPDH.

### Quantification of GFP-LC3 puncta

Cells were transfected with GFP-LC3 plasmid in the presence or absence of autophagy inducers for GFP-LC3 formation assay. Under confocal microscopy, GFP-LC3 puncta were quantified by counting the percentage of cells that accumulated in the spots or vacuoles. The diffuse distribution of GFP-LC3 in the cytoplasm and nucleus is considered as a non-autophagic point, whereas the intense punctate GFP-LC3 aggregates without nuclear GFP-LC3 are classified as autophagic points.

### mRFP-GFP-LC3 reporter assay

The mRFP-GFP-LC3 reporter gene detection takes advantage of the fact that GFP fluorescence is more sensitive to the acidic environment in autophagic lysosomes than mRFP fluorescence. Both GFP and mRFP are detected in autophagosomes and present as yellow puncta (GFP+/RFP+). Once autophagosomes and lysosomes fuse, GFP fluorescence will disappear due to acid lysosomal protease degradation of GFP, resulting in LC3 puncta only emitting RFP fluorescence indicating autolysosomes (GFP-/RFP+, Red puncta). Therefore, the dynamic transition from yellow spots to red puncta can indicate the process of functional autophagy flux. Twenty-four hours after plating, the designated cells were transfected with mRFP-GFP-LC3 plasmid, and then the cells were treated with Baf-A1 or control solvent. The cells were then fixed with 4% paraformaldehyde, and the nuclei were counterstained with DAPI (blue). Autophagy was determined by quantifying the percentage of cells with LC3 positive puncta, each condition was repeated three times, and at least 100 cells were counted.

### Clone formation

Cells were inoculated in a six-well plate at a density of 1000 cells per well, with 3 replicates for each group, and placed in an incubator and let stand. After 2 weeks, the medium was discarded, and the cells were fixed by Methanol for 30 min, stained with crystal violet staining solution, counted, and photographed under the microscope. The number of cells was greater than 50 as a clone, according to the clone size (cell number 50–100, 100–200, > 200), the clones were classified and counted.

### Xenograft tumor studies

The luciferase plasmid-labeled cells were treated accordingly, and then prepared into a cell suspension with a density of 1 × 10^7^/ml. Nude mice aged 8–12 weeks were injected subcutaneously with 100 μl of the cell suspension into the left armpit. The tumor growth was measured every week, and the animal imaging system was used to take pictures. After 40 days, nude mice were killed, and the tumors were removed, fixed with paraformaldehyde, and embedded for subsequent experiments.

### Cell proliferation and viability assay

Cells were seeded in 96-well plates (1000 cells/well) under specified conditions. After incubation for the corresponding time, CCK8 was added. Then cells were incubated for 1–2 h, and the absorbance at 540 nm was read on a microplate reader.

### Glutamine and glucose measurements

The cells were seeded into a six-well plate, and after changing to a fresh medium for 6–9 h, the levels of glutamine, ammonia, glucose and lactate in the medium were measured with a BioProfile FLEX analyzer (Nova Biomedical), and normalized to the number of cells in each well.

### RNA-seq library construction

RNA-seq was performed by Shanghai JingZhou Gene Biotechnology (Shanghai, China). Total RNA was isolated from three replicate samples of SIRT4 stably overexpressed human pancreatic cancer cells and control cells using TRIzol reagent (Invitrogen). mRNA was enriched with magnetic beads with Oligo(dT), and the enriched mRNA was fragmented. The cDNA was synthesized using the fragmented mRNA as template. After purification, end-repair, and addition of base A and sequencing junction, the obtained cDNA was recovered by agarose gel electrophoresis for the target size fragment and PCR amplification, thus completing the whole library preparation. The constructed libraries were sequenced by illumina NovaSeq 6000 high-throughput sequencing platform, and the obtained raw image data files were transformed into raw sequenced sequences (Sequenced Reads) by Base Calling analysis after image processing for subsequent analysis.

### RNA-sequencing analysis

Tophat v2.0.9 was used to map the cleaned reads to the human hg38 reference genome with two mismatches. Differentially expressed genes (DEGs) were identified using Cuffdiff. The p-value significance threshold in multiple tests was set by the false discovery rate (FDR). The fold changes (FCs) were also estimated according to the FPKM in each sample. The DEGs were selected using the filter criteria as follows: FDR ≤ 0.05 and FC ≥ 2.

### Mass spectrometric metabolomics analysis

Six replicates of SIRT4 stably overexpressed human pancreatic cancer cell lines and control cell lines were prepared, and the obtained samples were washed overnight in a speed vac and then homogenized in PBS (pH 7.4) using an Omni International bead homogenizer device at 6.45 m/s for 30 s. Homogenate was obtained using CHCl_3_: MeOH (2:1) to separate polar and nonpolar metabolites. Samples were centrifuged at 3000 rpm for 5 min and then analyzed with LC-MS/MS using the selected reaction monitoring (SRM) method with positive/negative ion polarity switching to a Xevo TQ-S mass spectrometer. The peak areas integrated using MassLynx 4.1 (Waters Inc.) have been normalized to the respective protein concentrations and the resulting peak areas have been subjected to relative quantification analysis using Metaboanalyst 3.0 (www.metaboanalyst.ca). The metabolites with variable importance in the projection (VIP) > 1 and *p* < 0.05 (student *t* test) were considered as significantly changed metabolites.

### Proteomics technology

Three pancreatic tissue samples each from SIRT4^−/−^ mice and wild-type mice were prepared. Appropriate amounts of tissue samples were weighed into a mortar pre-cooled with liquid nitrogen and well ground to powder with liquid nitrogen. Four volumes of lysis buffer (8 M urea, 1% protease inhibitor cocktail) were added to the cell powder and the samples were sonicated three times on ice using a high-intensity ultrasound processor (Scientz) in lysis buffer (8 M urea, 1% protease inhibitor cocktail). The remaining debris was removed and the protein concentration was determined. Trypsin was added to the resulting protein samples and the trypsinized peptides were desalted with Strata X C18 (Phenomenex) as well as vacuum freeze-dried. Peptides were lysed with 0.5 M TEAB and labeled according to the TMT kit operating instructions. The labeled peptides were graded by high pH inverse HPLC on an Agilent 300 Extend C18 column and separated by an UHPLC system before being injected into an NSI ion source for ionization and then analyzed by Q Exactive Plus mass spectrometry. Secondary mass spectrometry data were retrieved using Maxquant (v1.5.2.8).

For each category proteins, InterPro (a resource that provides functional analysis of protein sequences by classifying them into families and predicting the presence of domains and important sites) database was researched and a two-tailed Fisher’s exact test was employed to test the enrichment of the differentially expressed protein against all identified proteins. Protein domains with a corrected p-value < 0.05 were considered significant.

### Statistical analysis

Each experiment was performed at least three times. The sample size for all other graphs was indicated as “n = x” above the graphs. If the standard error of all randomly collected individual data points in a group is significantly smaller than the average value, the corresponding sample size is appropriate and creditable. Statistical analysis was performed using GraphPad Prism 8 software (GraphPad Software, San Diego, CA). Data were expressed as mean ± standard deviation (SD). For normally distributed data, A Student’s *t* test (two-sided) was used to compare two groups affected by one single variable. For non-normally distributed continuous variables, the nonparametric Kruskal–Wallis ranking sum test was used. Survival differences were determined using the Kaplan–Meier method and the Log-rank test. *p* values < 0.05 were considered statistically significant. All *p* values are indicated in the graphs (*p < 0.05; **p < 0.01; ***p < 0.001; ****p < 0.0001; n.s. not significant). This study selects a representative experimental result from three or more independent experiments to present.

## Supplementary information

Supplementary figure legends

Figure S1

Figure S2

Figure S3

Figure S4

Figure S5

Figure S6

Figure S7

Supplementary Table 1

Supplementary Table 2

Original picture of western boltting

Checklist

## Data Availability

The RNA-seq data were uploaded to GEO (GSE201110), the proteomic and metabolomic profiles were provided in Supplementary Table 2.

## References

[1] Kim KH, Lee MS (2014). Autophagy–a key player in cellular and body metabolism. Nat Rev Endocrinol..

[2] Levine B, Kroemer G (2019). Biological functions of autophagy genes: a disease perspective. Cell.

[3] Galluzzi L, Green DR (2019). Autophagy-independent functions of the autophagy machinery. Cell.

[4] Amaravadi RK, Kimmelman AC, Debnath J (2019). Targeting autophagy in cancer: recent advances and future directions. Cancer Discov.

[5] Wellenstein MD, Coffelt SB, Duits DEM, van Miltenburg MH, Slagter M, de Rink I (2019). Loss of p53 triggers WNT-dependent systemic inflammation to drive breast cancer metastasis. Nature.

[6] Levine AJ (2020). p53: 800 million years of evolution and 40 years of discovery. Nat Rev Cancer..

[7] Duffy MJ, Synnott NC, O’Grady S, Crown J (2020). Targeting p53 for the treatment of cancer. Semin Cancer Biol..

[8] Amit M, Takahashi H, Dragomir MP, Lindemann A, Gleber-Netto FO, Pickering CR (2020). Loss of p53 drives neuron reprogramming in head and neck cancer. Nature.

[9] Tang Q, Su Z, Gu W, Rustgi AK (2020). Mutant p53 on the path to metastasis. Trends Cancer.

[10] Khan H, Reale M, Ullah H, Sureda A, Tejada S, Wang Y (2020). Anti-cancer effects of polyphenols via targeting p53 signaling pathway: updates and future directions. Biotechnol Adv.

[11] Crighton D, Wilkinson S, O’Prey J, Syed N, Smith P, Harrison PR (2006). DRAM, a p53-induced modulator of autophagy, is critical for apoptosis. Cell.

[12] Goiran T, Duplan E, Rouland L, El Manaa W, Lauritzen I, Dunys J (2018). Nuclear p53-mediated repression of autophagy involves PINK1 transcriptional down-regulation. Cell Death Differ.

[13] Tasdemir E, Maiuri MC, Galluzzi L, Vitale I, Djavaheri-Mergny M, D’Amelio M (2008). Regulation of autophagy by cytoplasmic p53. Nat Cell Biol.

[14] Herzig S, Shaw RJ (2018). AMPK: guardian of metabolism and mitochondrial homeostasis. Nat Rev Mol Cell Biol.

[15] Pineda CT, Ramanathan S, Fon Tacer K, Weon JL, Potts MB, Ou YH (2015). Degradation of AMPK by a cancer-specific ubiquitin ligase. Cell.

[16] Lin SC, Hardie DG (2018). AMPK: sensing glucose as well as cellular energy status. Cell Metab.

[17] Garcia D, Shaw RJ (2017). AMPK: mechanisms of cellular energy sensing and restoration of metabolic balance. Mol Cell.

[18] Faubert B, Boily G, Izreig S, Griss T, Samborska B, Dong Z (2013). AMPK is a negative regulator of the Warburg effect and suppresses tumor growth in vivo. Cell Metab.

[19] Shackelford DB, Shaw RJ (2009). The LKB1-AMPK pathway: metabolism and growth control in tumour suppression. Nat Rev Cancer..

[20] Fu L, Dong Q, He J, Wang X, Xing J, Wang E (2017). SIRT4 inhibits malignancy progression of NSCLCs, through mitochondrial dynamics mediated by the ERK-Drp1 pathway. Oncogene.

[21] Li T, Li Y, Liu T, Hu B, Li J, Liu C (2020). Mitochondrial PAK6 inhibits prostate cancer cell apoptosis via the PAK6-SIRT4-ANT2 complex. Theranostics.

[22] Miyo M, Yamamoto H, Konno M, Colvin H, Nishida N, Koseki J (2015). Tumour-suppressive function of SIRT4 in human colorectal cancer. Br J Cancer.

[23] Wang YS, Du L, Liang X, Meng P, Bi L, Wang YL (2019). Sirtuin 4 depletion promotes hepatocellular carcinoma tumorigenesis through regulating adenosine-monophosphate-activated protein kinase alpha/mammalian target of rapamycin axis in mice. Hepatology.

[24] Anderson KA, Huynh FK, Fisher-Wellman K, Stuart JD, Peterson BS, Douros JD (2017). SIRT4 is a lysine deacylase that controls leucine metabolism and insulin secretion. Cell Metab.

[25] Zaganjor E, Vyas S, Haigis MC (2017). SIRT4 is a regulator of insulin secretion. Cell Chem Biol.

[26] Steinley D (2006). Profiling local optima in K-means clustering: developing a diagnostic technique. Psychol. Methods.

[27] White E, Mehnert JM, Chan CS (2015). Autophagy, metabolism, and cancer. Clin Cancer Res.

[28] Kimmelman AC, White E (2017). Autophagy and tumor metabolism. Cell Metab.

[29] Chang HC, Guarente L (2014). SIRT1 and other sirtuins in metabolism. Trends Endocrinol Metab.

[30] Zhu S, Dong Z, Ke X, Hou J, Zhao E, Zhang K (2019). The roles of sirtuins family in cell metabolism during tumor development. Semin Cancer Biol.

[31] Jeong SM, Xiao C, Finley LW, Lahusen T, Souza AL, Pierce K (2013). SIRT4 has tumor-suppressive activity and regulates the cellular metabolic response to DNA damage by inhibiting mitochondrial glutamine metabolism. Cancer Cell.

[32] Csibi A, Fendt SM, Li C, Poulogiannis G, Choo AY, Chapski DJ (2013). The mTORC1 pathway stimulates glutamine metabolism and cell proliferation by repressing SIRT4. Cell.

[33] Deshpande V, Castillo CF, Muzikansky A, Deshpande A, Zukerberg L, Warshaw AL (2004). Cytokeratin 19 is a powerful predictor of survival in pancreatic endocrine tumors. Am J Surg Pathol.

[34] Maiuri MC, Galluzzi L, Morselli E, Kepp O, Malik SA, Kroemer G (2010). Autophagy regulation by p53. Curr Opin Cell Biol.

[35] Rosenfeldt MT, O’Prey J, Morton JP, Nixon C, MacKay G, Mrowinska A (2013). p53 status determines the role of autophagy in pancreatic tumour development. Nature.

[36] Boya P, Reggiori F, Codogno P (2013). Emerging regulation and functions of autophagy. Nat Cell Biol.

[37] Du L, Liu X, Ren Y, Li J, Li P, Jiao Q (2020). Loss of SIRT4 promotes the self-renewal of Breast Cancer Stem Cells. Theranostics.

[38] Li Z, Li H, Zhao Z-B, Zhu W, Feng P-P, Zhu X-W (2019). SIRT4 silencing in tumor-associated macrophages promotes HCC development via PPARδ signalling-mediated alternative activation of macrophages. J Exp Clin Cancer Res.

[39] Lang A, Anand R, Altinoluk-Hambuchen S, Ezzahoini H, Stefanski A, Iram A (2018). Correction: SIRT4 interacts with OPA1 and regulates mitochondrial quality control and mitophagy. Aging.

[40] He L, Wang J, Yang Y, Zou P, Xia Z, Li J (2022). SIRT4 suppresses doxorubicin-induced cardiotoxicity by regulating the AKT/mTOR/autophagy pathway. Toxicology.

[41] Shaw E, Talwadekar M, Rashida Z, Mohan N, Acharya A, Khatri S (2020). Anabolic SIRT4 exerts retrograde control over TORC1 signaling by glutamine sparing in the mitochondria. Mol Cel Biol..

[42] Levine B, Abrams J (2008). p53: the Janus of autophagy?. Nat Cell Biol.

[43] Tang J, Di J, Cao H, Bai J, Zheng J (2015). p53-mediated autophagic regulation: a prospective strategy for cancer therapy. Cancer Lett.

[44] Hardie DG, Schaffer BE, Brunet A (2016). AMPK: an energy-sensing pathway with multiple inputs and outputs. Trends Cell Biol.

[45] He G, Zhang YW, Lee JH, Zeng SX, Wang YV, Luo Z (2014). AMP-activated protein kinase induces p53 by phosphorylating MDMX and inhibiting its activity. Mol Cell Biol.

