# Supplementary material for: Sirtuin 4 activates autophagy and inhibits tumorigenesis by upregulating the p53 signaling pathway
Source: Cell Death Differ. 2022 Oct 8;30(2):313–26. doi: 10.1038/s41418-022-01063-3 (PMC9950374; doi:10.1038/s41418-022-01063-3)
Supplement: Supplementary file 1 — Supplementary figure legends [file 41418_2022_1063_MOESM1_ESM.docx]

**Supplementary Figure Annotations**

**Supplementary Figure 1**

A-E. Scatter plots showing correlation of transcript expression between SIRT1, SIRT3-7 and autophagy pathway in pancreatic cancer.

**Supplementary Figure 2**

A. Western Blot detection of SIRT4 expression in normal pancreatic tissue and pancreatic cancer tissue.

B. Representative images of IHC staining showing SIRT4 expression in pancreas sections from normal tissue, PanINs and PDAC, respectively.

C. Western blot detection showing the expression level changes of the SIRT4 in pancreatic tissues of WT, KC and KPC mice.

D. Representative images of immunohistochemical (IHC) detection showing the expressions of SIRT4 in pancreatic tissues of WT, KC and KPC mice.

E. Western blot detection showing the expression levels of SIRT4 in pancreatic cancer cells and normal pancreatic epithelial cells.

F. MTT assay to detect the effects of SIRT4 overexpression or knockdown on the proliferation of pancreatic cancer cells.

G. Colony formation assay to detect the effects of SIRT4 overexpression or knockdown on the colony-forming abilities of pancreatic cancer cells.

H. Representative images (left) and its quantification (right panel) of the autophagic flux detection with the mRFP-GFP-LC3 reporter in HPDE6-C7 cells co-transfected with scramble or sh-SIRT4 vectors, respectively, with or w/o Baf-A1 treatment.

I. SEM was applied to detect autophagy in HPDE6-C7 cells described above. Red arrows indicate autophagosomes or autolysosomes.

J. Representative images (left) and its quantification (right panel) of the autophagic flux detection with the mRFP-GFP-LC3 reporter in HS766T cells co-transfected with control, SIRT4, or SIRT4H161Y vectors, respectively, with or w/o Baf-A1 treatment. K. SEM was applied to detect autophagy in HS766T cells described above. Red arrows indicate autophagosomes or autolysosomes.

L. Western blot analysis to detect the expression level of autophagy-related proteins in HPDE6-C7 cells transfected with scramble or sh-SIRT4 vectors, with or w/o Baf-A1 treatment.

M. Western blot analysis to detect the expression level of autophagy-related proteins in HS766T cells transfected with control, or SIRT4 vectors, with or w/o Baf-A1 treatment.

SPSS software was used for independent sample t test, (* P<0.05; ** P<0.01; *** P<0.001).

**Supplementary Figure 3**

A. Representative images of IHC staining to detect the expression level of p-p53 and its downstream proteins in pancreatic tissues of wild-type mice and SIRT4^-/-^ mice.

B-E. Cell metabolism was observed in pancreatic cancer cell lines with stable SIRT4 overexpression and stable knockout; SPSS software was used for independent sample t test, (* P<0.05; ** P<0.01; *** P<0.001).

**Supplementary Figure 4**

A. Representative images (left) and its quantification (right panel) of the autophagic flux detection with the mRFP-GFP-LC3 reporter in HS766T cells co-transfected with control, SIRT4, or SIRT4H161Y vectors, respectively, in the absence or presence of pifithrin-α.

B. Representative images (left) and its quantification (right panel) of the autophagic flux detection with the mRFP-GFP-LC3 reporter in HPDE6-C7 cells co-transfected with scramble or sh-SIRT4 vectors, respectively, in the absence or presence of Inauhzin.

C. Activate the p53 signaling pathway in pancreatic cancer cell lines knocked down by SIRT4, and observe the status of LC3-GFP in the cells.

D-E. Representative ventral view images of bioluminescence from nude mice subcutaneously injected with Capan-2 (D) and CCC-HPE-2 (E) cells.

F. Quantification of bioluminescence of tumors in anesthetized nude mice with indicated treatments.

G. Tumor weight are shown with indicated treatments. SPSS software was used for independent sample t test, (* P<0.05; ** P<0.01; *** P<0.001).

**Supplementary Figure 5**

A-B. After treatment of AMPK activator in CCC-HPE-2 and HPDE6-C7 cells with stable SIRT4 knockdown, qRT-PCR was used to detect the expression level of autophagy-related genes.

C-D. After knocking down AMPK expression in SIRT4-overexpressing pancreatic cancer cell lines, qRT-PCR was used to detect the expression of autophagy related genes.

**Supplementary Figure 6**

A. Representative images (left) and its quantification (right panel) of the autophagic flux detection with the mRFP-GFP-LC3 reporter in HPDE6-C7 cells co-transfected with scramble or sh-SIRT4 vectors, respectively, in the absence or presence of platycodin D, an AMPK activator. The indicated cells were treated with or without 10 nM Baf-A1 inhibited autophagosome-lysosome fusion.

B. After AMPK was depleted in Hs766T cell lines overexpressing SIRT4, the mRFP-GFP-LC3 dual fluorescence labeling method was applied to detect the occurrence of autophagy. The indicated cells were treated with or without 10 nM Baf-A1 inhibited autophagosome-lysosome fusion.

C. AMPK activator treated SIRT4 knockdown separately after low pancreatic cancer cells and control cell lines, observe the LC3-GFP in the cells.

D. After knocking down AMPK expression in pancreatic cancer cell lines overexpressing SIRT4, observe the LC3-GFP in the cells.

SPSS software was used for independent sample t test, (* P<0.05; ** P<0.01).

**Supplementary Figure 7**

A-B. Representative images (A) and its quantification (B) of sphere formation assay to detect the tumorigenic ability of CCC-HPE-2 and HPDE6-C7 cells transfected with scramble or sh-SIRT4 vectors, respectively, in the response to AMPK overexpression. C-D. Representative images (C) and its quantification (D) of sphere formation assay to detect the tumorigenic ability of Capan-2 and Hs766T cells bearing control or SIRT4 vectors, respectively, in the absence or presence of Dorsomorphin, an AMPK inhibitor.

E-F. Representative images (E) and its quantification (F) of sphere formation assay to detect the tumorigenic ability of primary pancreatic cells from wildtype and SIRT4-/- mice in the response to AMPK overexpression.

G-H. Representative images (G) and its quantification (H) of sphere formation assay to detect the tumorigenic ability of primary pancreatic cells from wildtype and SIRT4-/- mice in the absence or presence of platycodin D.
