# Supplementary figures and images for: Sirtuin 4 activates autophagy and inhibits tumorigenesis by upregulating the p53 signaling pathway
Source: Cell Death Differ. 2022 Oct 8;30(2):313–26. doi: 10.1038/s41418-022-01063-3 (PMC9950374; doi:10.1038/s41418-022-01063-3)

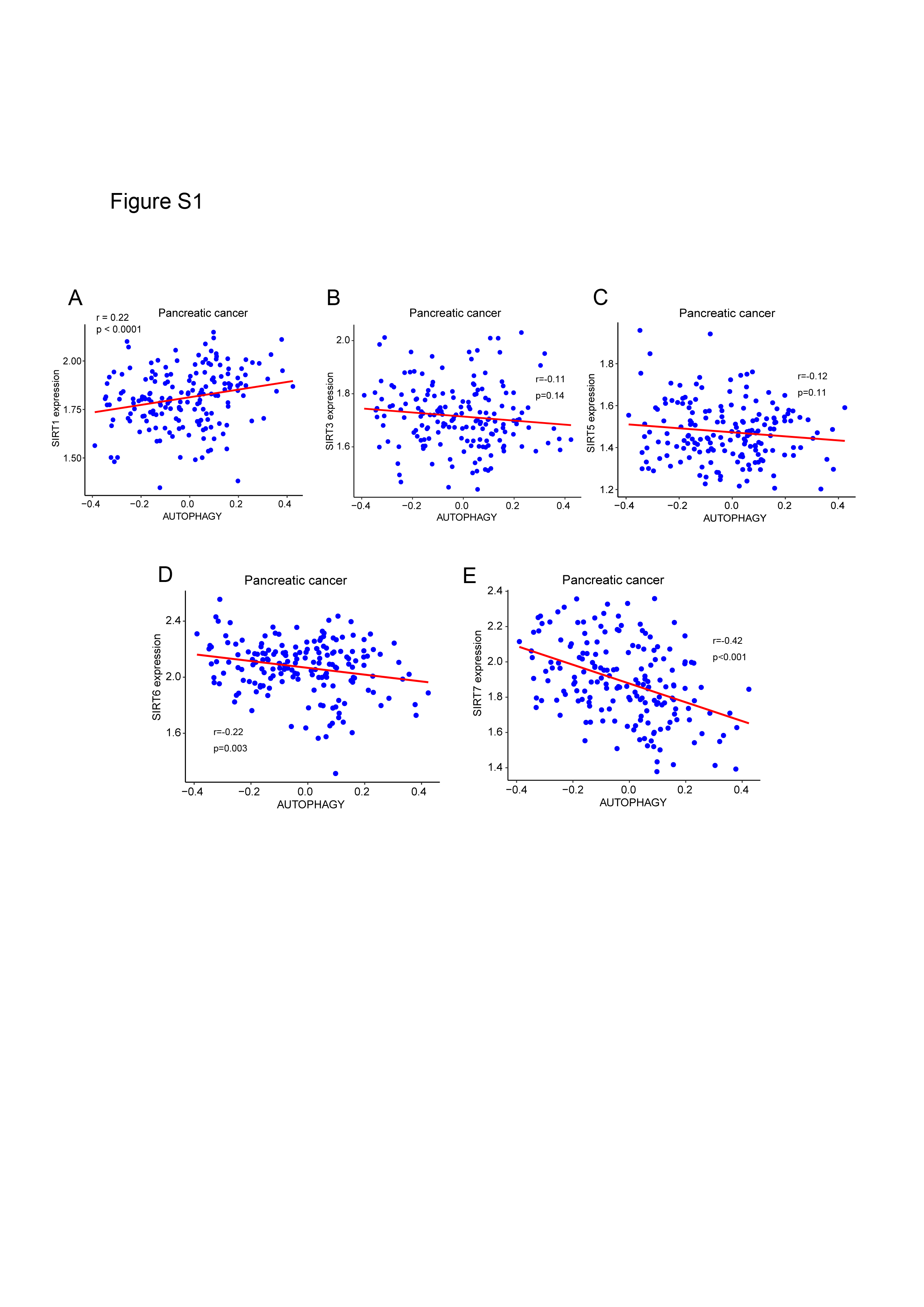

Supplement: Supplementary file 2 — Figure S1 [file 41418_2022_1063_MOESM2_ESM.tif]

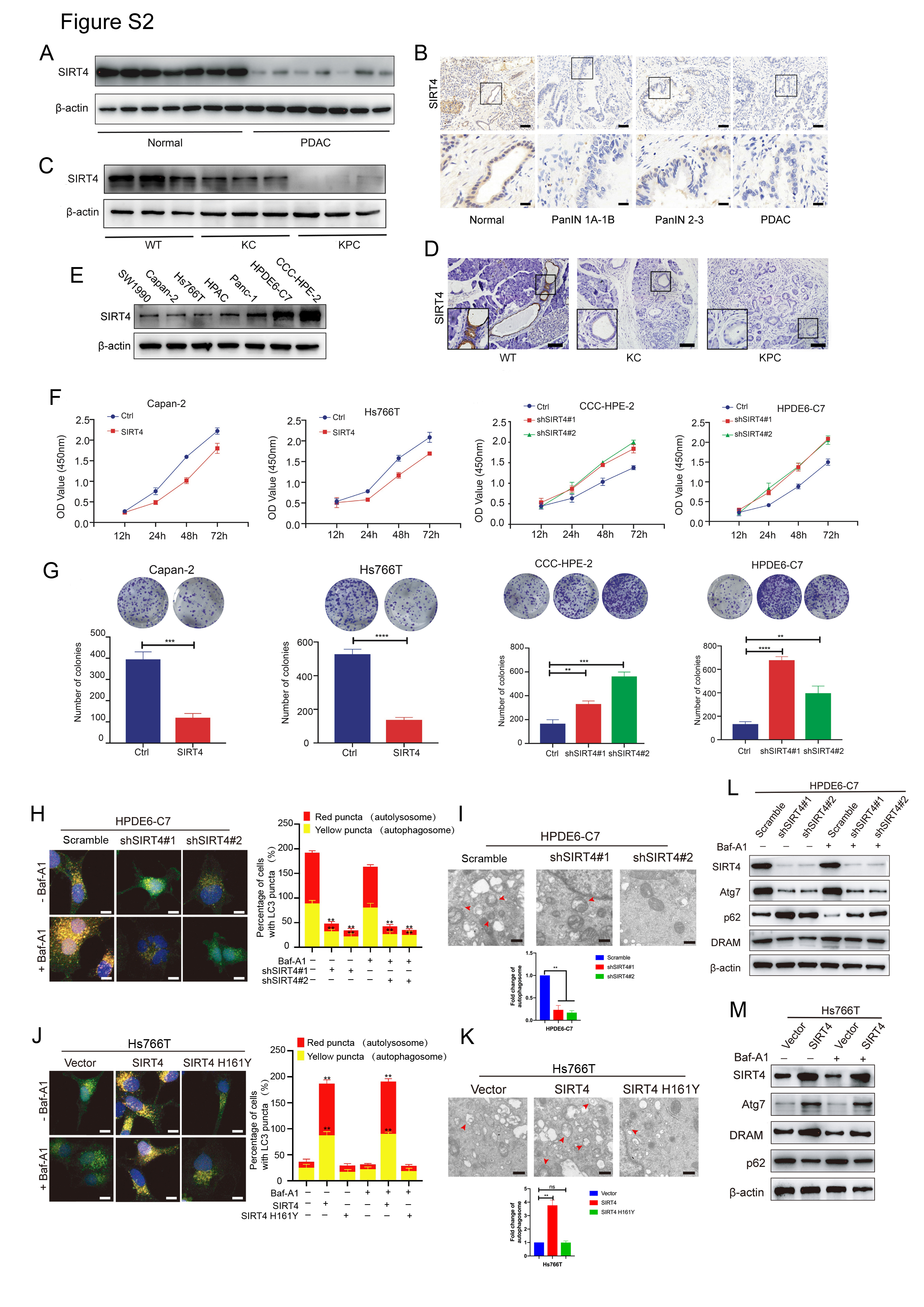

Supplement: Supplementary file 3 — Figure S2 [file 41418_2022_1063_MOESM3_ESM.tif]

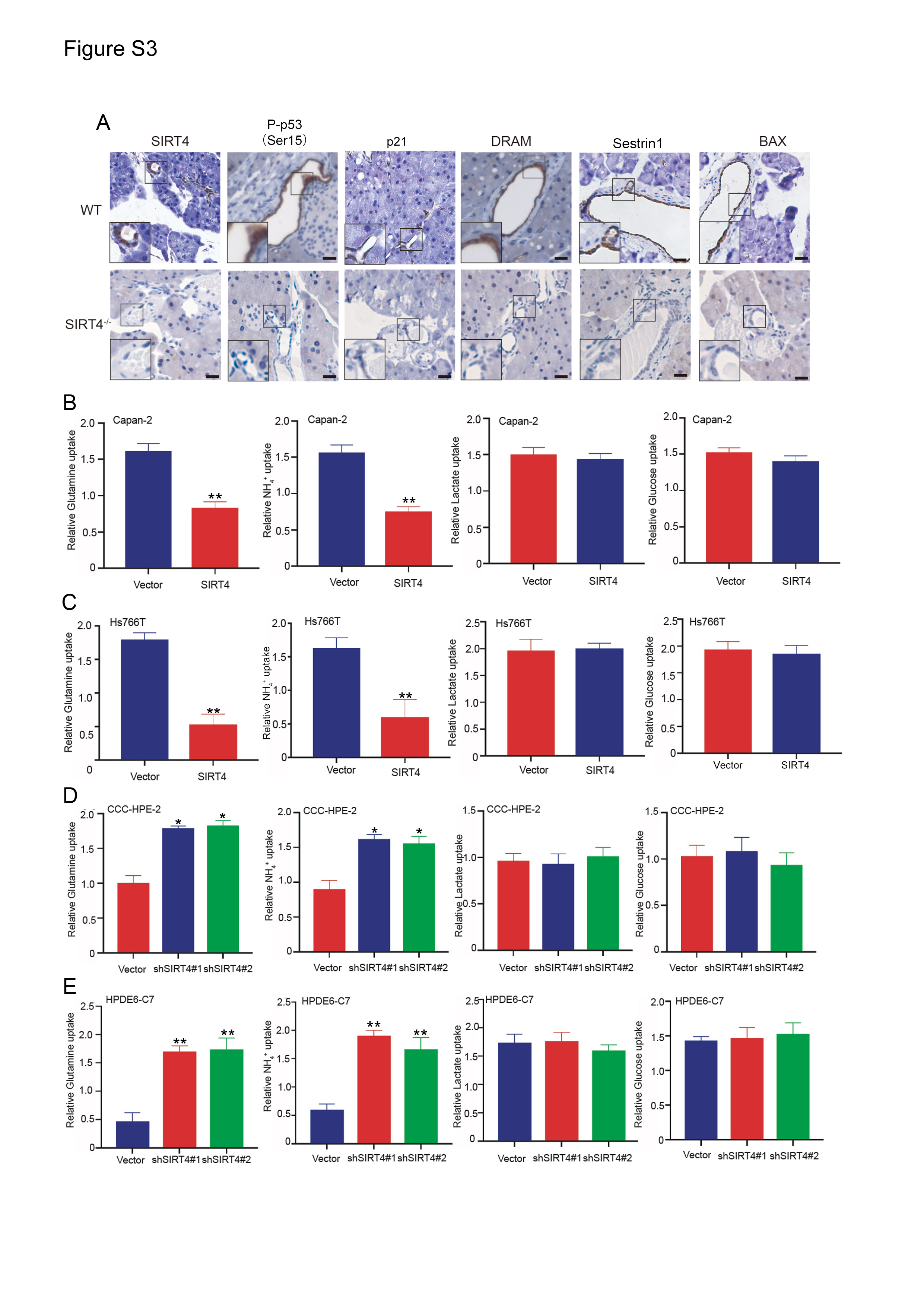

Supplement: Supplementary file 4 — Figure S3 [file 41418_2022_1063_MOESM4_ESM.tif]

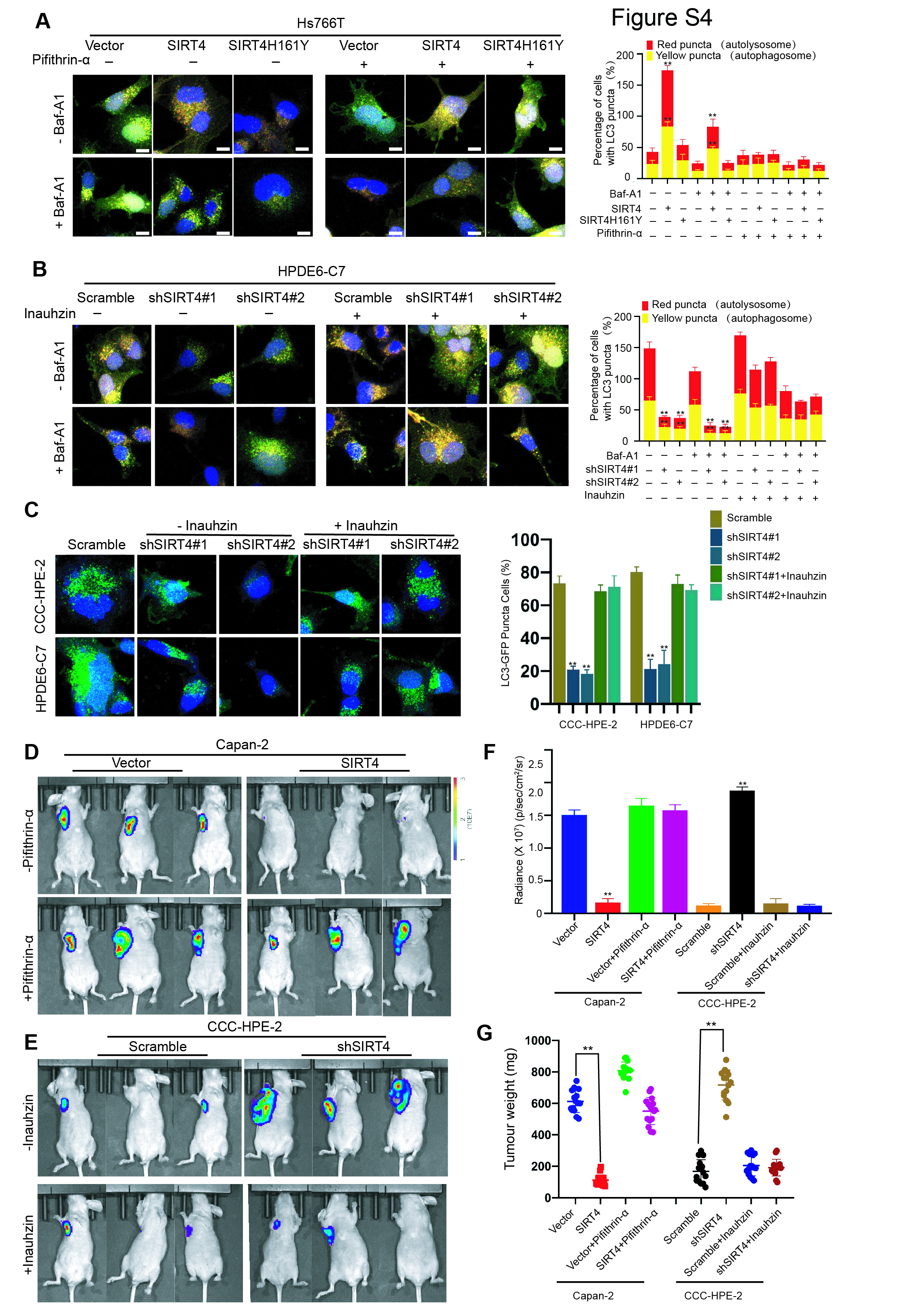

Supplement: Supplementary file 5 — Figure S4 [file 41418_2022_1063_MOESM5_ESM.tif]

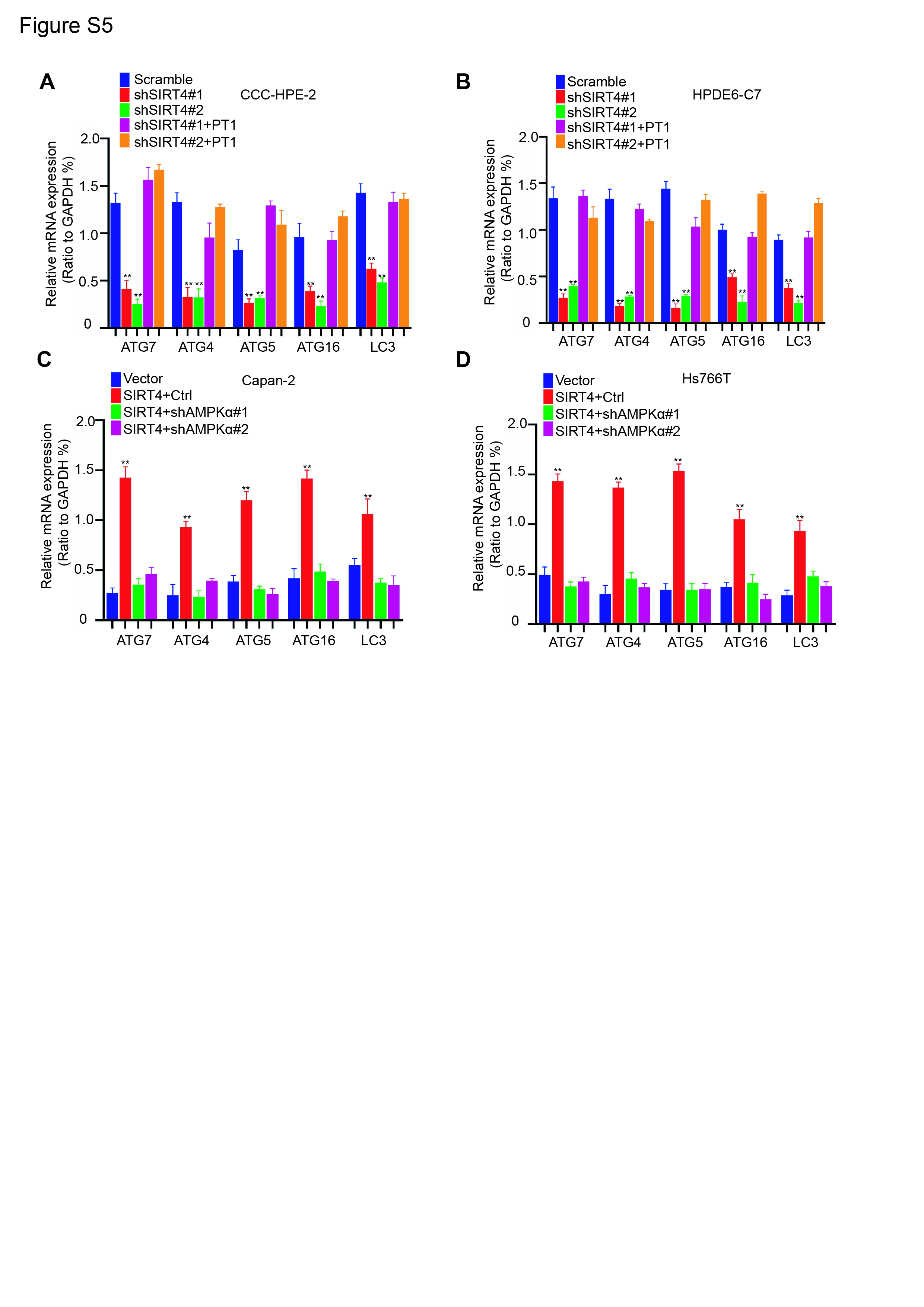

Supplement: Supplementary file 6 — Figure S5 [file 41418_2022_1063_MOESM6_ESM.tif]

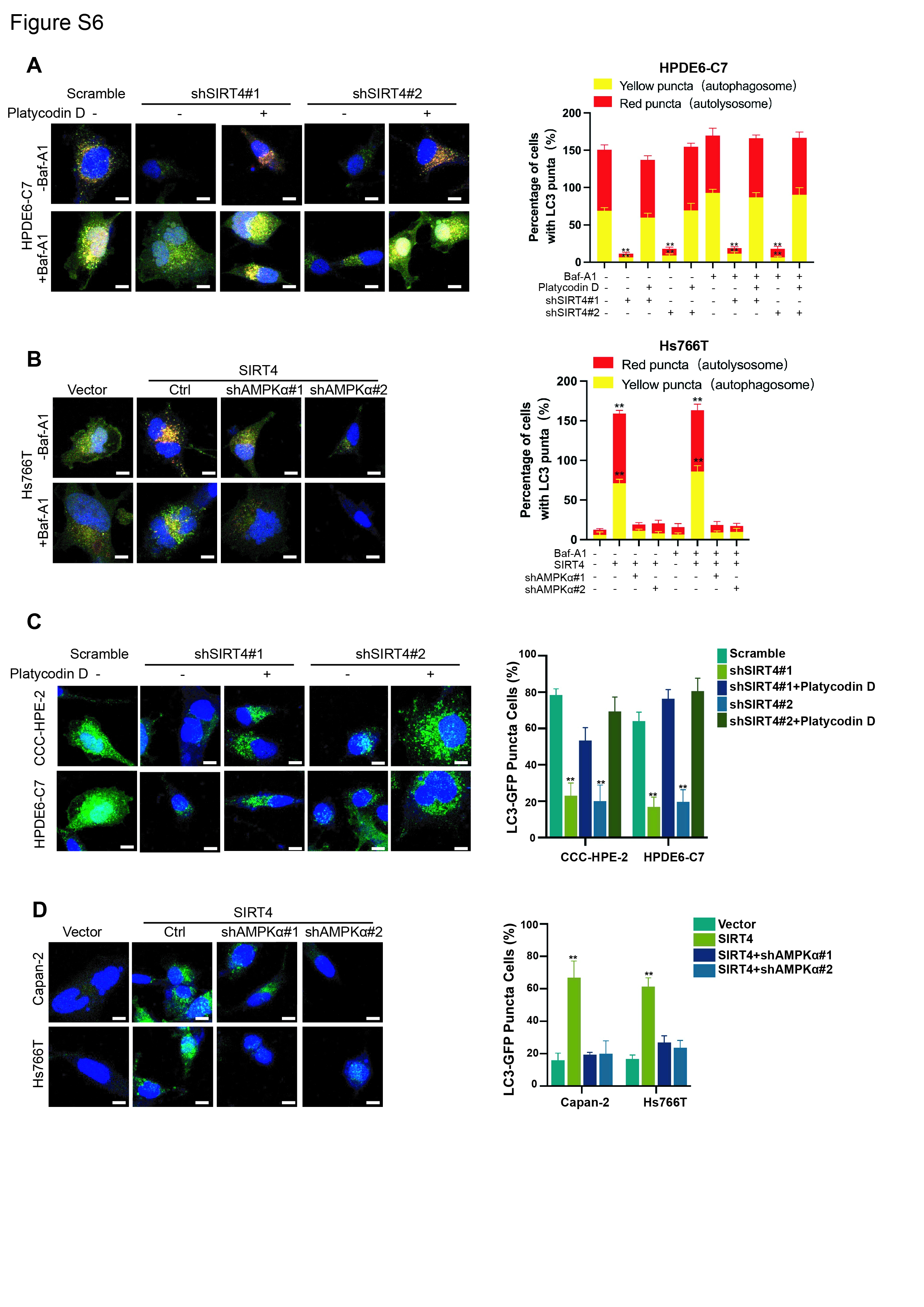

Supplement: Supplementary file 7 — Figure S6 [file 41418_2022_1063_MOESM7_ESM.tif]

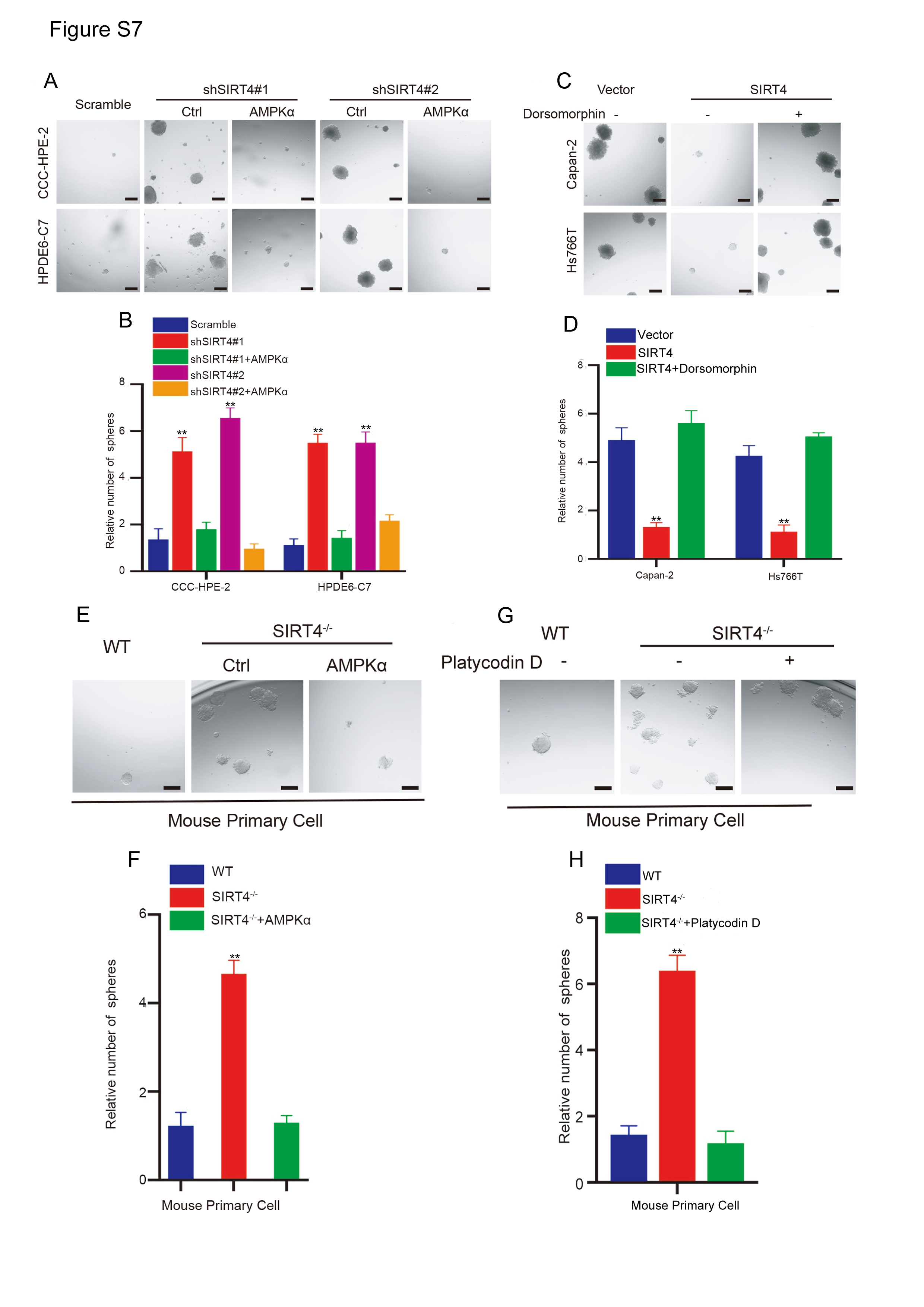

Supplement: Supplementary file 8 — Figure S7 [file 41418_2022_1063_MOESM8_ESM.tif]
