# Supplementary material for: Sirtuin 4 activates autophagy and inhibits tumorigenesis by upregulating the p53 signaling pathway
Source: Cell Death Differ. 2022 Oct 8;30(2):313–26. doi: 10.1038/s41418-022-01063-3 (PMC9950374; doi:10.1038/s41418-022-01063-3)
Supplement: Supplementary file 9 — Supplementary Table 1 [file 41418_2022_1063_MOESM9_ESM.docx]

**REAGENT or RESOURCE**

| REAGENT or RESOURCE | SOURCE | IDENTIFIER |
| --- | --- | --- |
| Antibodies |  |  |
| Mouse polyclonal anti-SIRT4 | Sigma | Cat# SAB1407208 |
| Rabbit polyclonal anti-SIRT4 | Sigma | Cat# SAB2108304 |
| Rabbit monoclonal anti-Phospho-p53 (Ser15) | Cell Signaling Technologies | Cat #9284 |
| Rabbit monoclonal anti-Phospho-p53 (Ser15) | Abcam | Cat #ab223868 |
| Mouse monoclonal anti-p53 | Cell Signaling Technologies | Cat # 2524 |
| Rabbit monoclonal anti-p21 | Cell Signaling Technologies | Cat # 37543 |
| Rabbit monoclonal anti-p21 | Cell Signaling Technologies | Cat # 2947 |
| Rabbit monoclonal anti-Bcl-2 | Cell Signaling Technologies | Cat #3498 |
| Mouse monoclonal anti-LC3B | Cell Signaling Technologies | Cat # 83506 |
| Rabbit monoclonal anti-SQSTM1/p62 | Cell Signaling Technologies | Cat # 23214S |
| Mouse monoclonal anti-SQSTM1/p62 | Cell Signaling Technologies | Cat # 88588 |
| Rabbit monoclonal anti-Bax | Cell Signaling Technologies | Cat # 14796S |
| Rabbit monoclonal anti-Bax | Cell Signaling Technologies | Cat # 5023 |
| Rabbit Polyclonal anti-DRAM | Sigma | Cat # PRS4035 |
| Rabbit monoclonal anti-SESN1 | Abcam | Cat # ab134091 |
| Mouse polyclonal anti-SESN1 | Abcam | Cat # ab67156 |
| Mouse monoclonal anti-β-actin | Cell Signaling Technologies | Cat # 4970S |
| Rabbit monoclonal anti-Atg7 | Abcam | Cat # ab133528 |
| Rabbit monoclonal anti-Phospho-AMPKα | Cell Signaling Technologies | Cat # 50081S |
| Rabbit monoclonal anti-AMPKα | Cell Signaling Technologies | Cat # 5831S |
| Rabbit monoclonal anti-mTOR | Cell Signaling Technologies | Cat # 2983S |
| Rabbit monoclonal anti-Phospho-mTOR | Cell Signaling Technologies | Cat # 5536S |
| Rabbit monoclonal anti-Acetyl-CoA Carboxylase | Cell Signaling Technologies | Cat # 3676S |
| Rabbit monoclonal Phospho-Acetyl-CoA Carboxylase | Cell Signaling Technologies | Cat# 11818S |
| Chemicals |  |  |
| Bafilomycin A1 | MedChemExpress | Cat# 88899-55-2 |
| RIPA buffer | Beyotime Biotechnology | Cat# P0013E |
| DAPI | Solarbio | Solarbio |
| DMEM | GIBCO | Cat# C11995500BT |
| Fetal Bovine Serum | GIBCO | Cat# 10100 |
| Lipofectamine 2000 | Thermo Fisher Scientific | Cat# 11668027 |
| Polybrene | Millipore | Cat# TR-1003-G |
| Rapamycin, mTOR inhibitor | Solarbio | Cat # R8140 |
| Pifithrin-α, p53 inhibitor | Selleck | Cat #S2929 |
| Inauhzin, p53 activator | Selleck | Cat #S6744 |
| Platycodin D, AMPKα activator | MCE | Cat #HY-N1411 |
| Dorsomorphin, AMPKα inhibitor | MCE | Cat # HY-13418A |
| Invitrogen TRIzol | ThermoFisher | Cat# 15596026 |
| PrimeScript™ RT Master Mix | Takara | Cat# RR036A |
| TB Green® Fast qPCR Mix | Takara | Cat# RR430A |
| Experimental Models: Cell Lines |  |  |
| Human：Capan-2 | ATCC | HTB-80 |
| Human：Hs766T | ATCC | HTB-134 |
| Human：HPAC | ATCC | CRL-2119 |
| Human：SW1990 | ATCC | CRL-2172 |
| Human：HPDE6-C7 | Broad Institute | N/A |
| Human：PANC-1 | ATCC | CRL-1469 |
| Experimental Models: Mouse |  |  |
| SIRT4^flox / flox^ mice | Cyagen | N/A |
| PDX-cre mice | Cyagen | N/A |
| Lox-Stop-Lox Kras^G12D^ mice | Cyagen | N/A |
| Trp53^R172H^ mice | Cyagen | N/A |
| Mouse: C57BL/6 | Shan Dong University | N/A |
| Nude mice | Charles River | N/A |
| Biological Samples |  |  |
| Human PDAC Tissue | The Qilu Hospital of Shandong University | N/A |
| Software |  |  |
| Prism v8 | GraphPad | www.graphpad.com |
