# Supplementary material for: Sirtuin 4 activates autophagy and inhibits tumorigenesis by upregulating the p53 signaling pathway
Source: Cell Death Differ. 2022 Oct 8;30(2):313–26. doi: 10.1038/s41418-022-01063-3 (PMC9950374; doi:10.1038/s41418-022-01063-3)

Fig 2K

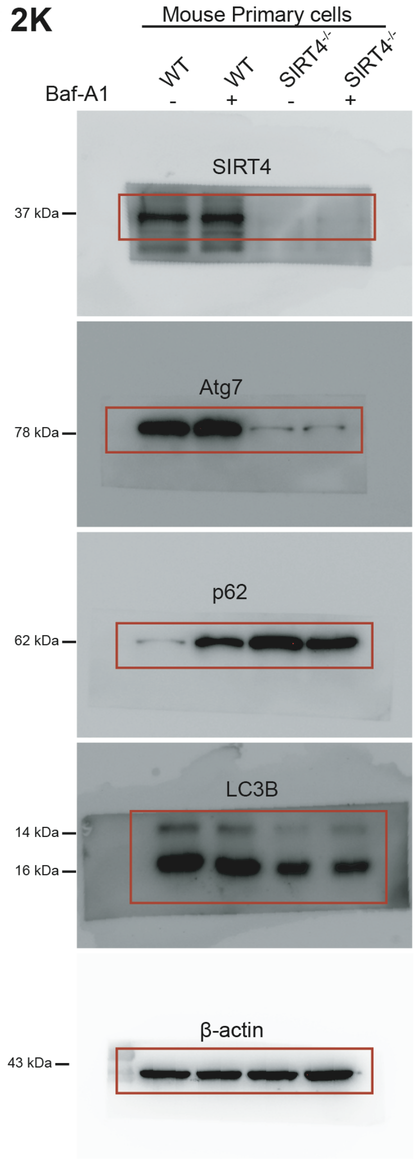

Fig 2L

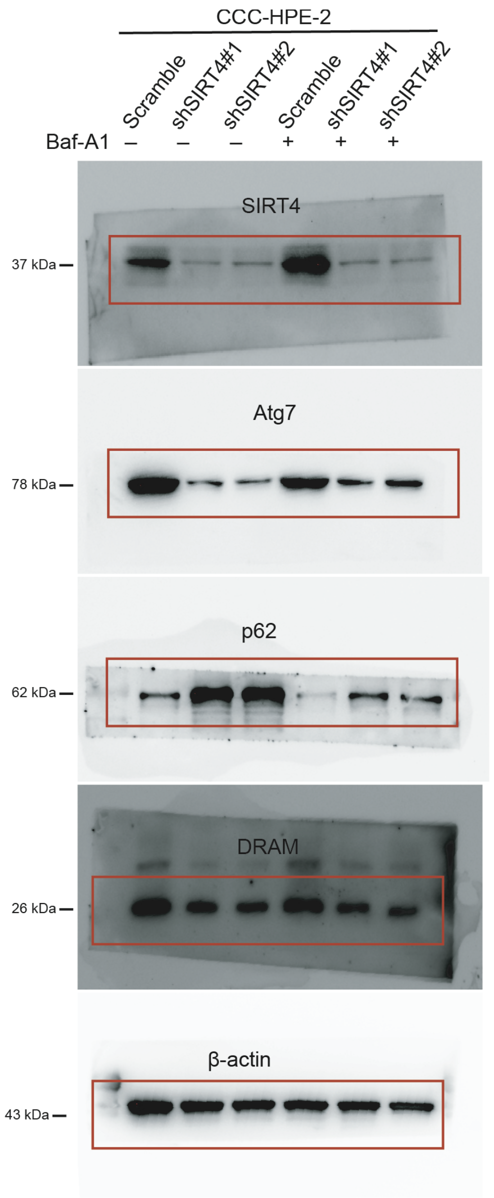

Fig 2M

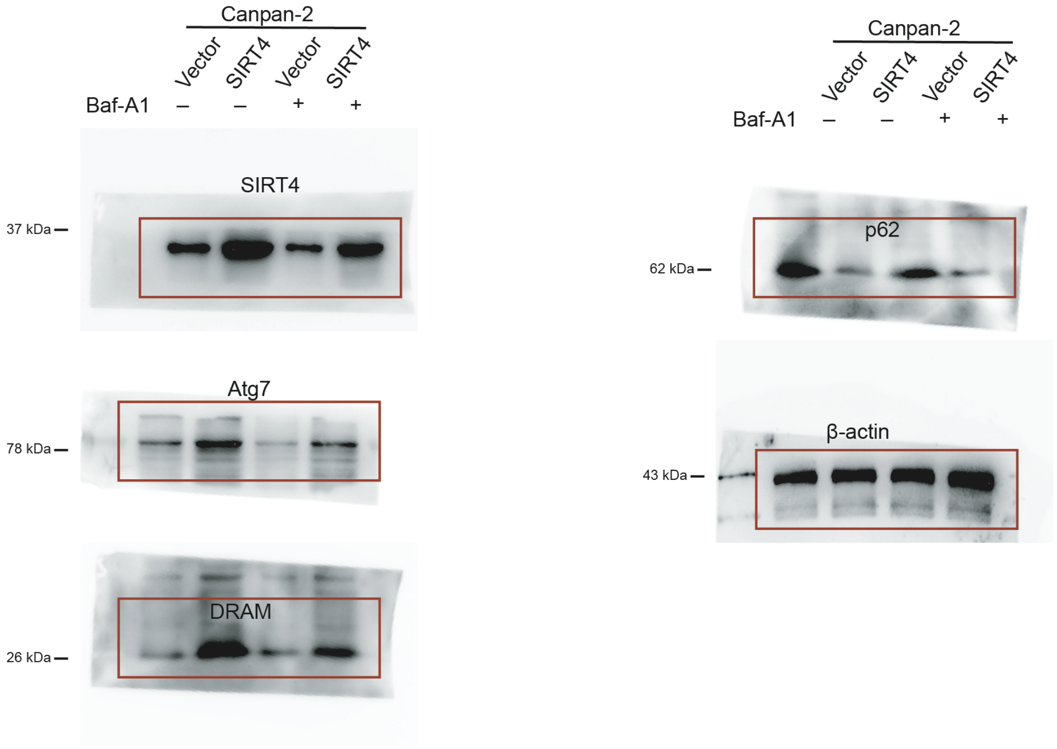

Fig 3C

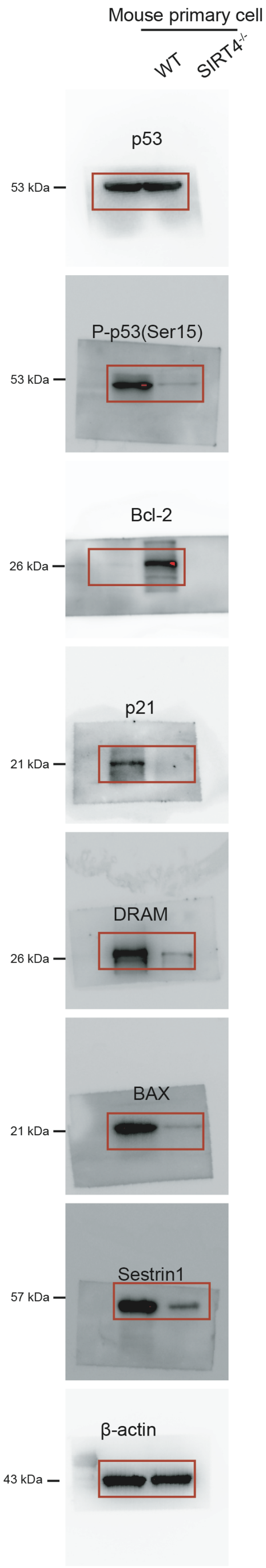

Fig 3D

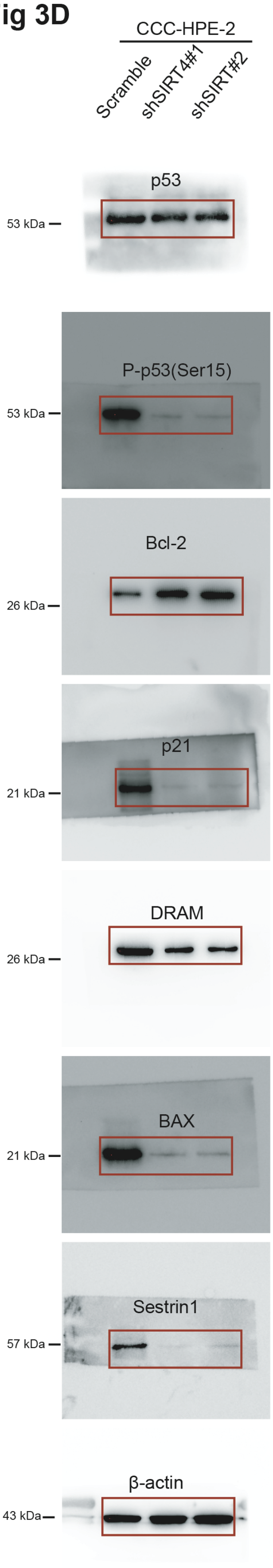

Fig 3E

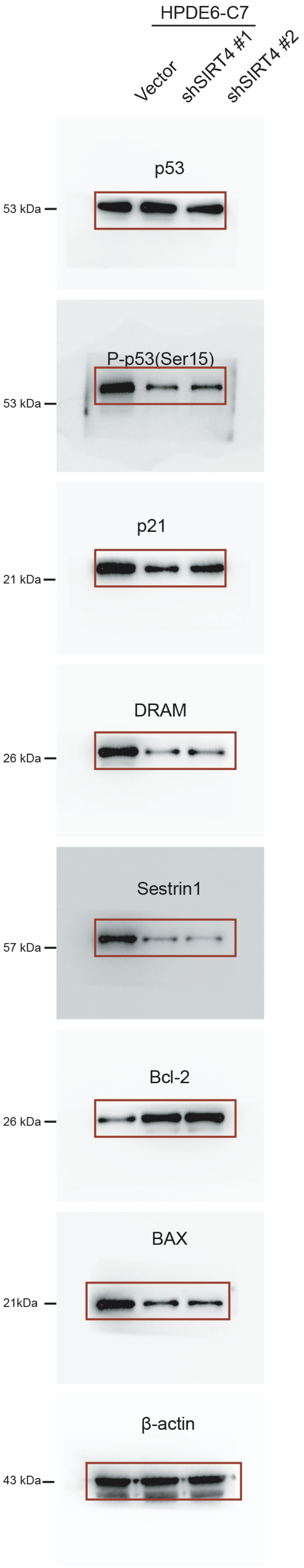

Fig 3F

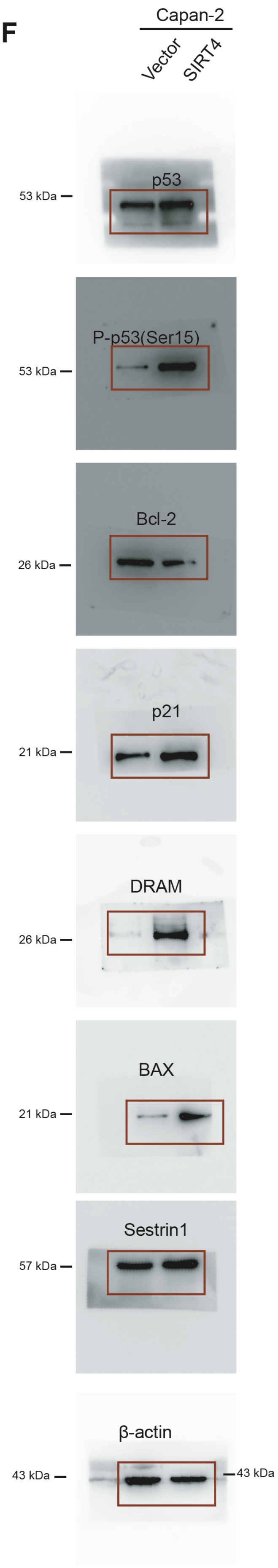
Capan-2
Vector
SIRT4
p53
53 kDa
P-p53(Ser15)
53 kDa
Bcl-2
26 kDa
p21
21 kDa
DRAM
26 kDa
BAX
21 kDa
Sestrin1
57 kDa
β-actin
43 kDa
43 kDa

Fig 3G

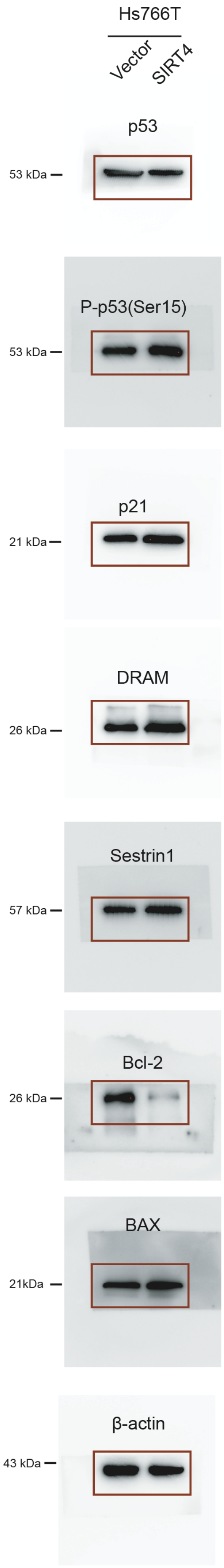
Hs766T
Vector
SIRT4
p53
53 kDa
P-p53(Ser15)
53 kDa
p21
21 kDa
DRAM
26 kDa
Sestrin1
57 kDa
Bcl-2
26 kDa
BAX
21kDa
β-actin
43 kDa

Fig 3L

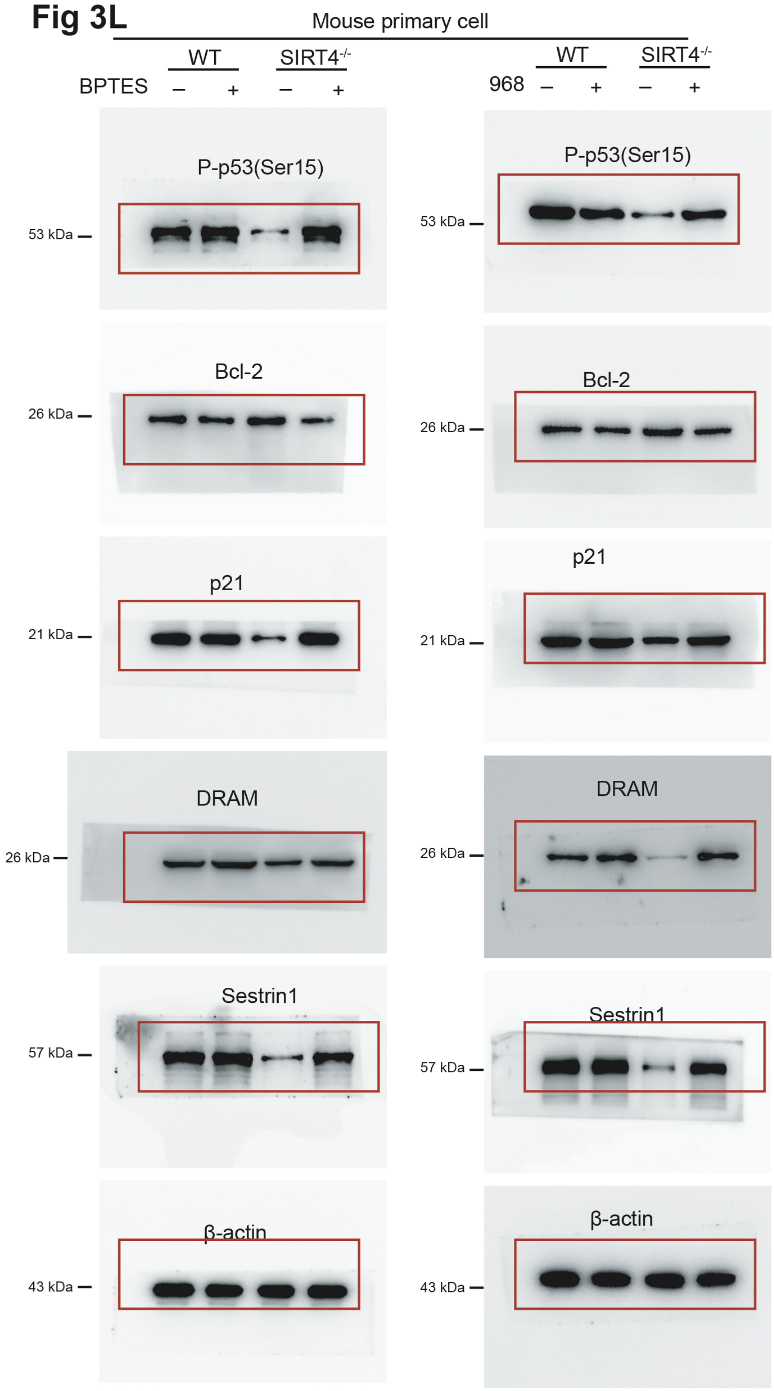

Mouse primary cell
WT
SIRT4-/-
BPTES
–
+
–
+
WT
SIRT4-/-
968
–
+
–
+
P-p53(Ser15)
53 kDa
Bcl-2
26 kDa
p21
21 kDa
DRAM
26 kDa
Sestrin1
57 kDa
β-actin
43 kDa

Fig 3M

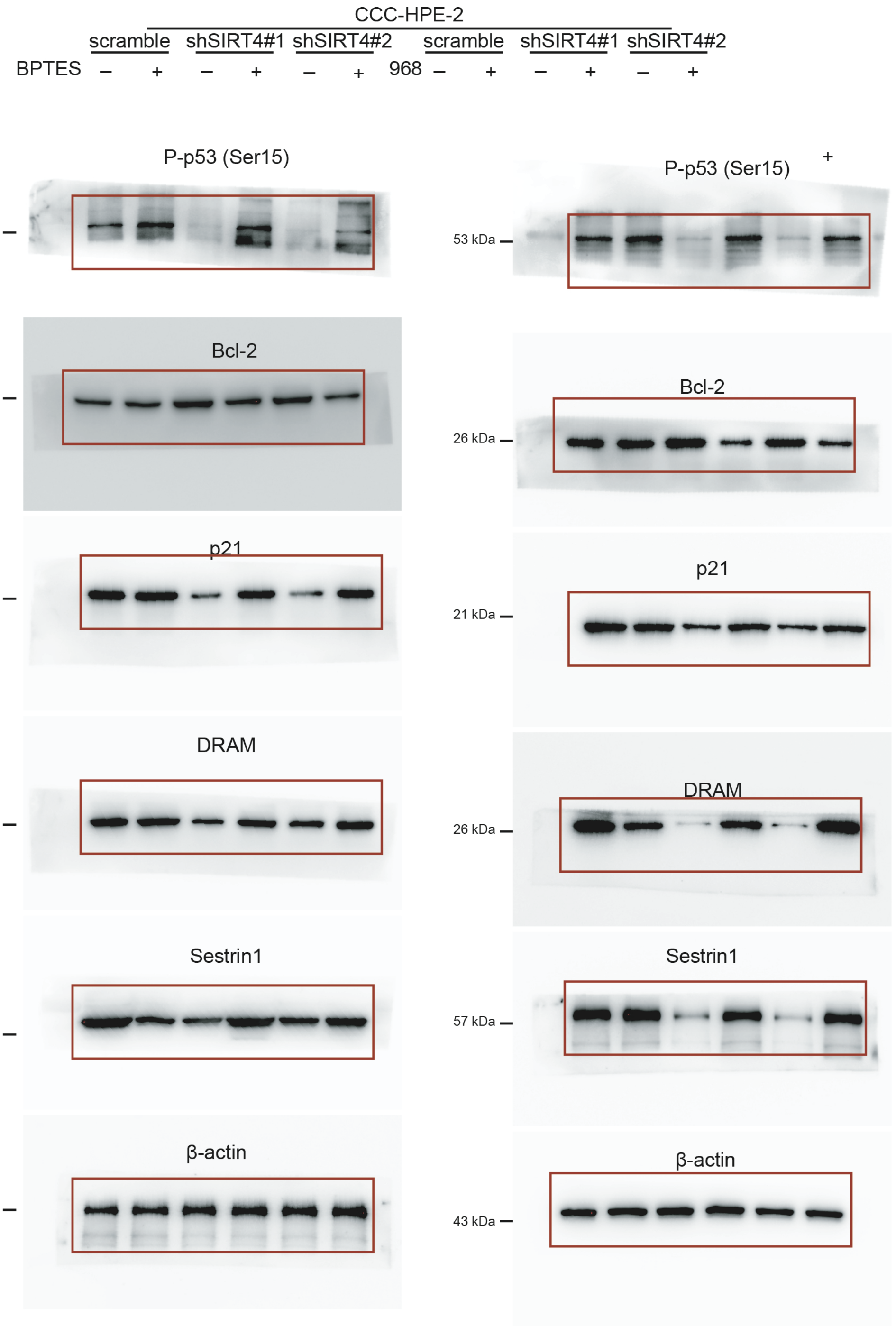
CCC-HPE-2
scramble
shSIRT4#1
shSIRT4#2
BPTES
968
P-p53 (Ser15)
53 kDa
Bcl-2
26 kDa
p21
21 kDa
DRAM
26 kDa
Sestrin1
57 kDa
β-actin
43 kDa

Fig 4D

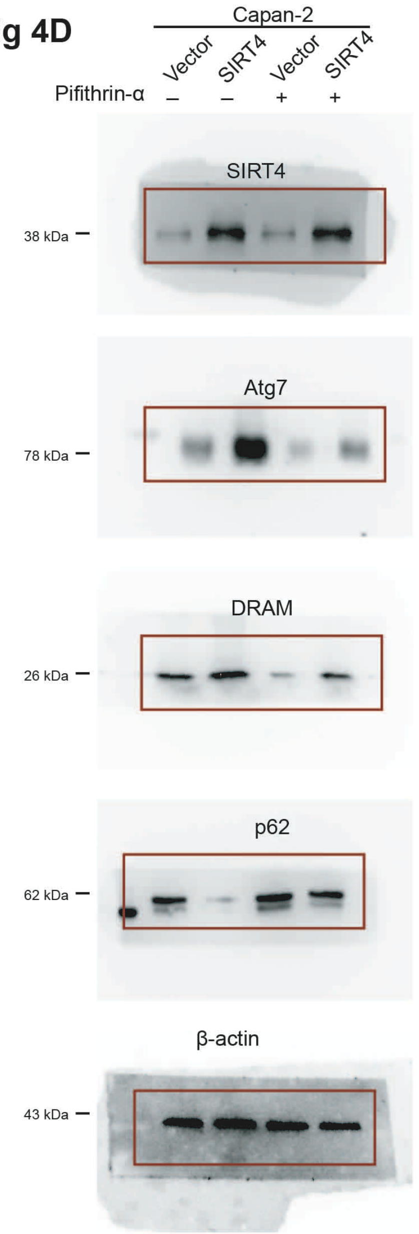
Capan-2
Vector
SIRT4
Vector
SIRT4
Pifithrin-α – – + +
SIRT4
38 kDa
Atg7
78 kDa
DRAM
26 kDa
p62
62 kDa
β-actin
43 kDa

Fig 4E

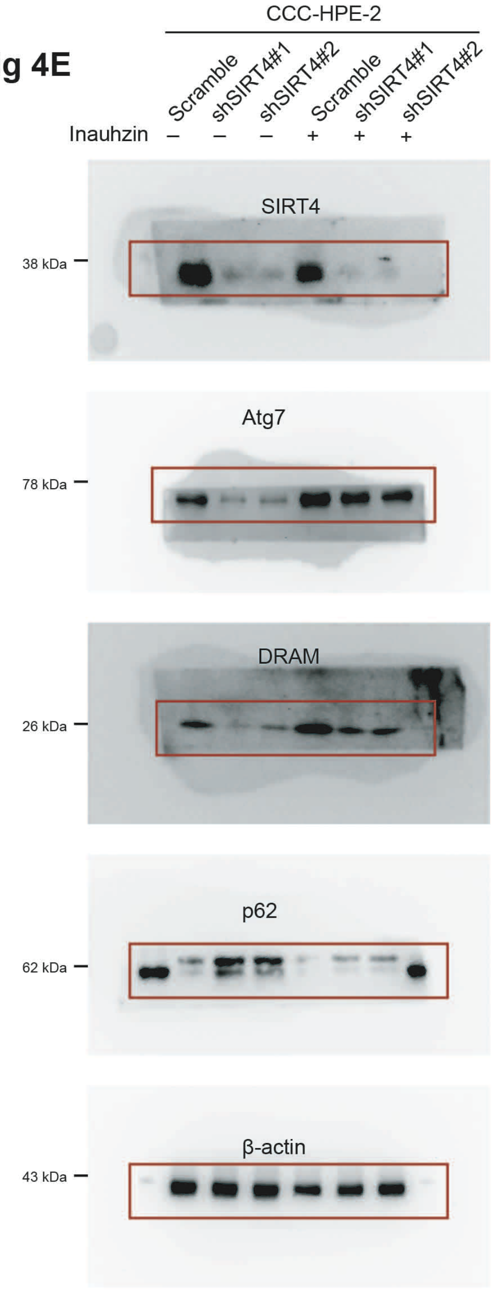
CCC-HPE-2
Scramble
shSIRT4#1
shSIRT4#2
Scramble
shSIRT4#1
shSIRT4#2
Inauhzin – – – + + +
SIRT4
38 kDa
Atg7
78 kDa
DRAM
26 kDa
p62
62 kDa
β-actin
43 kDa

Fig 4H

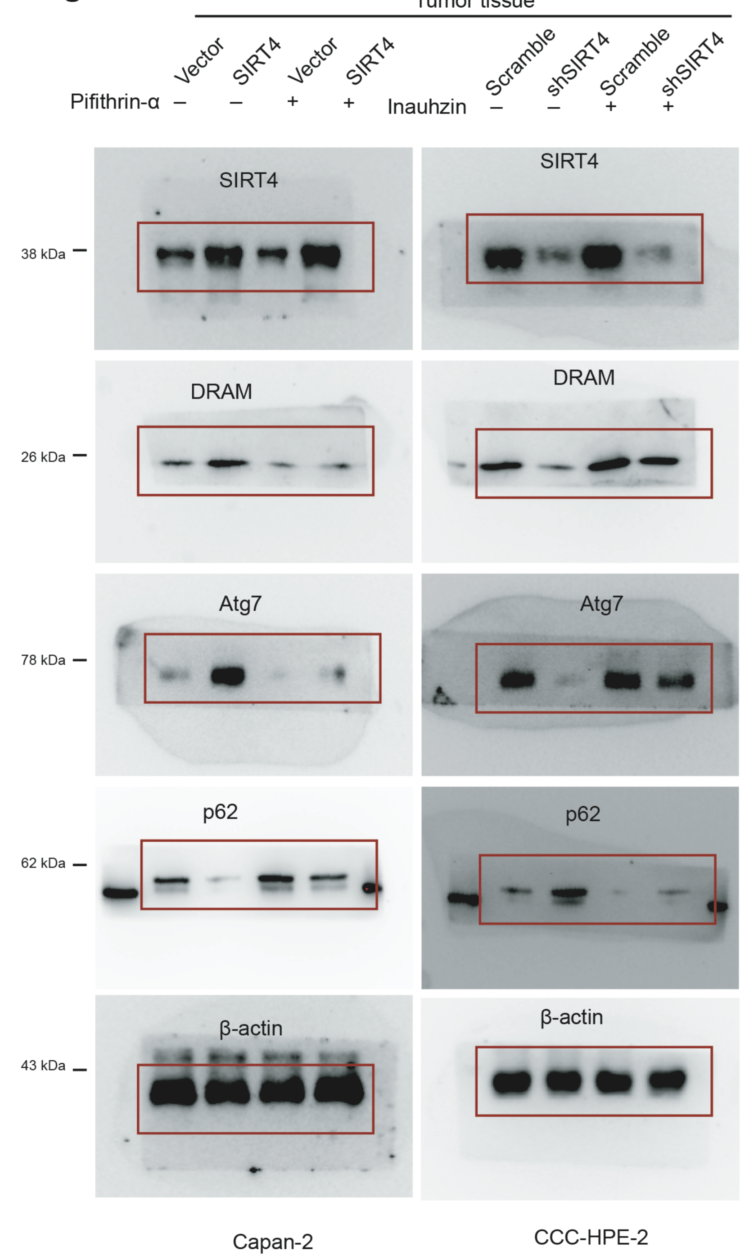

Tumor tissue
Vector
SIRT4
Vector
SIRT4
Scramble
shSIRT4
Scramble
shSIRT4
Pifithrin-α – – + +
Inauhzin – – + +
SIRT4
SIRT4
38 kDa
DRAM
DRAM
26 kDa
Atg7
Atg7
78 kDa
p62
p62
62 kDa
β-actin
β-actin
43 kDa
Capan-2
CCC-HPE-2

Fig 5A

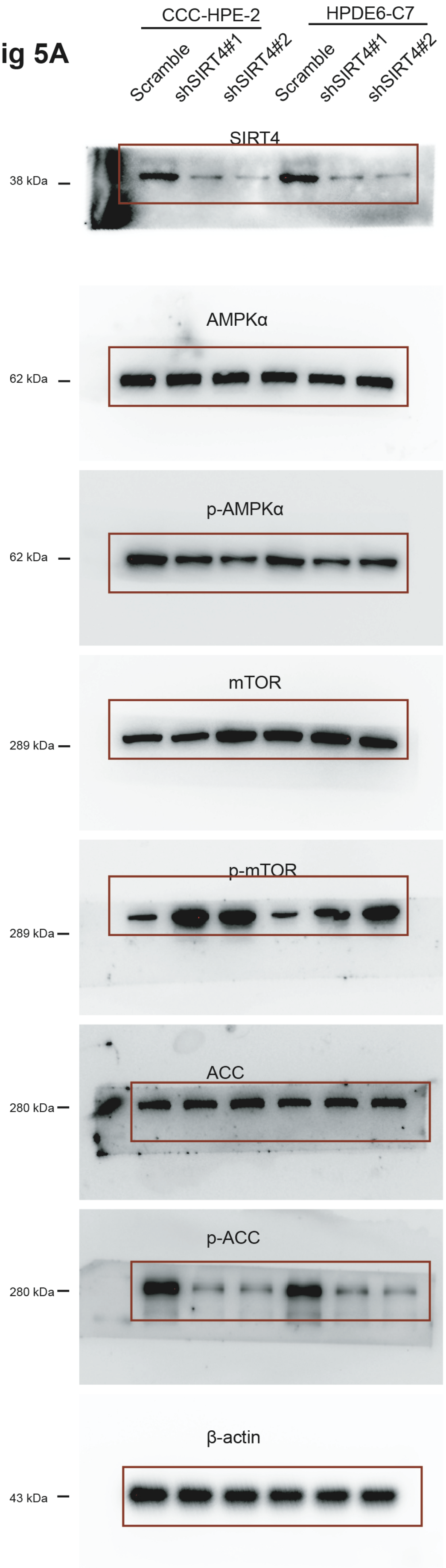

CCC-HPE-2
HPDE6-C7
Scramble
shSIRT4#1
shSIRT4#2
Scramble
shSIRT4#1
shSIRT4#2
SIRT4
38 kDa
AMPKα
62 kDa
p-AMPKα
62 kDa
mTOR
289 kDa
p-mTOR
289 kDa
ACC
280 kDa
p-ACC
280 kDa
β-actin
43 kDa

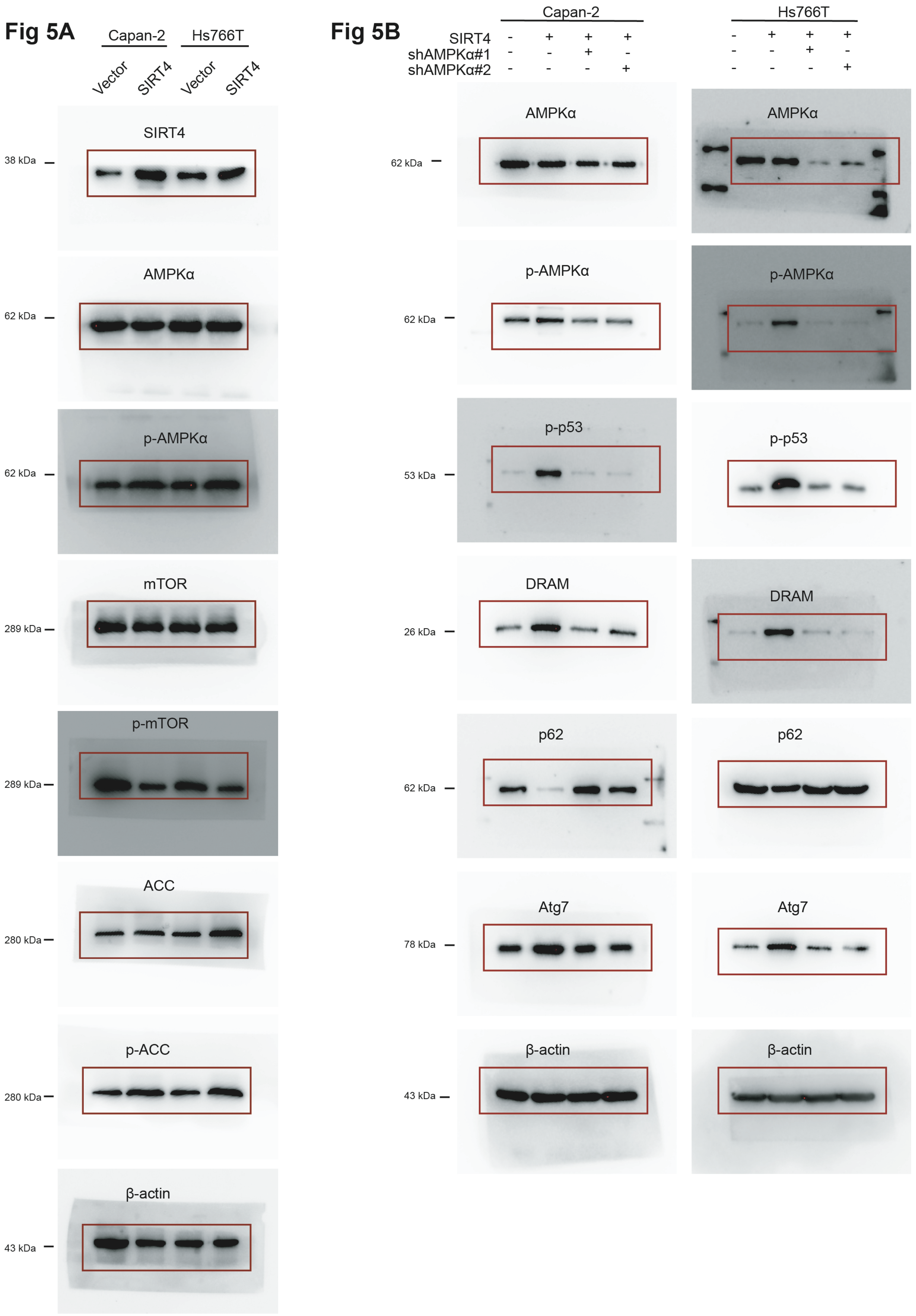
Fig 5A
Capan-2
Hs766T
Vector
SIRT4
Vector
SIRT4
SIRT4
38 kDa
AMPKα
62 kDa
p-AMPKα
62 kDa
mTOR
289 kDa
p-mTOR
289 kDa
ACC
280 kDa
p-ACC
280 kDa
β-actin
43 kDa
Fig 5B
Capan-2
Hs766T
SIRT4
shAMPKα#1
shAMPKα#2
AMPKα
62 kDa
p-AMPKα
62 kDa
p-p53
53 kDa
DRAM
26 kDa
p62
62 kDa
Atg7
78 kDa
β-actin
43 kDa

Fig 6H

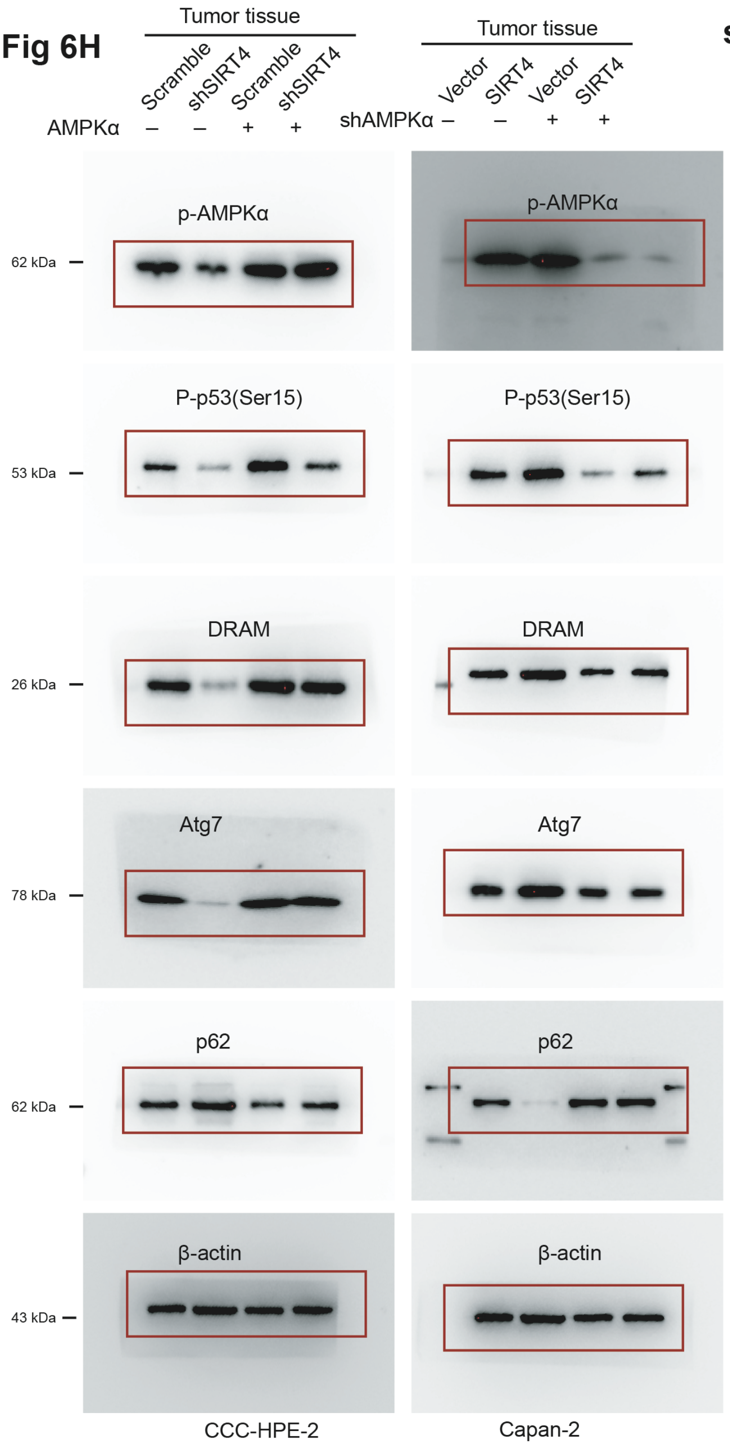

sFig 2C

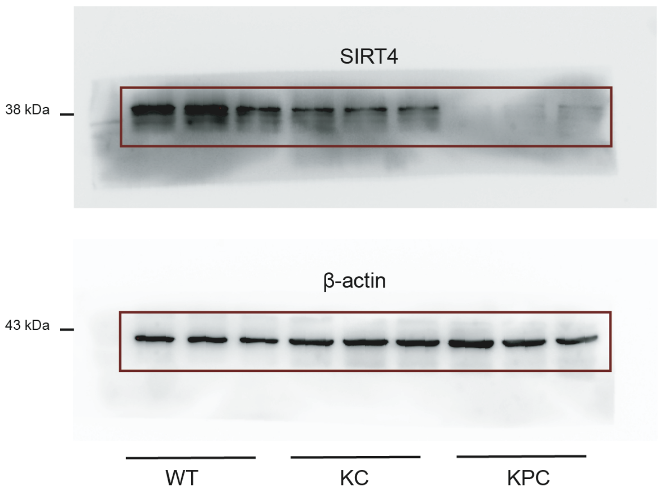

sFig 2E

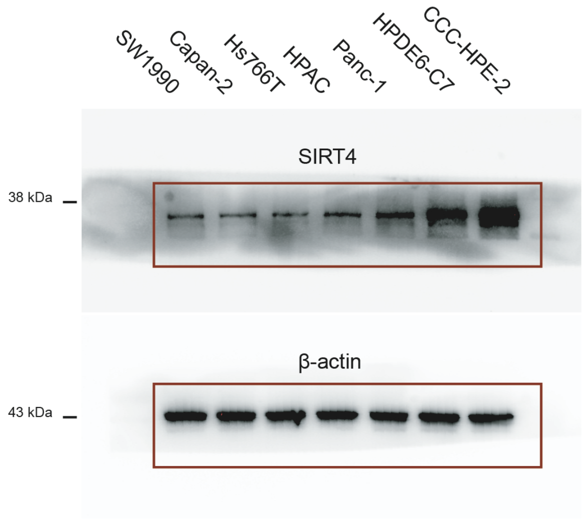

sFig 2A

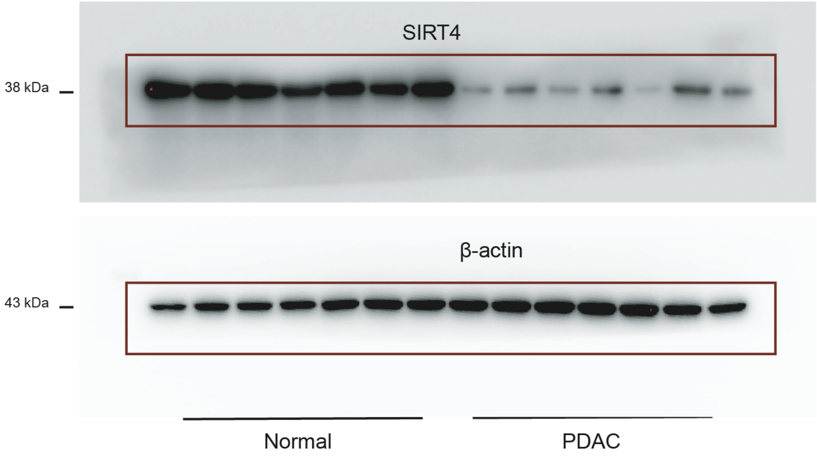

sFig 2L

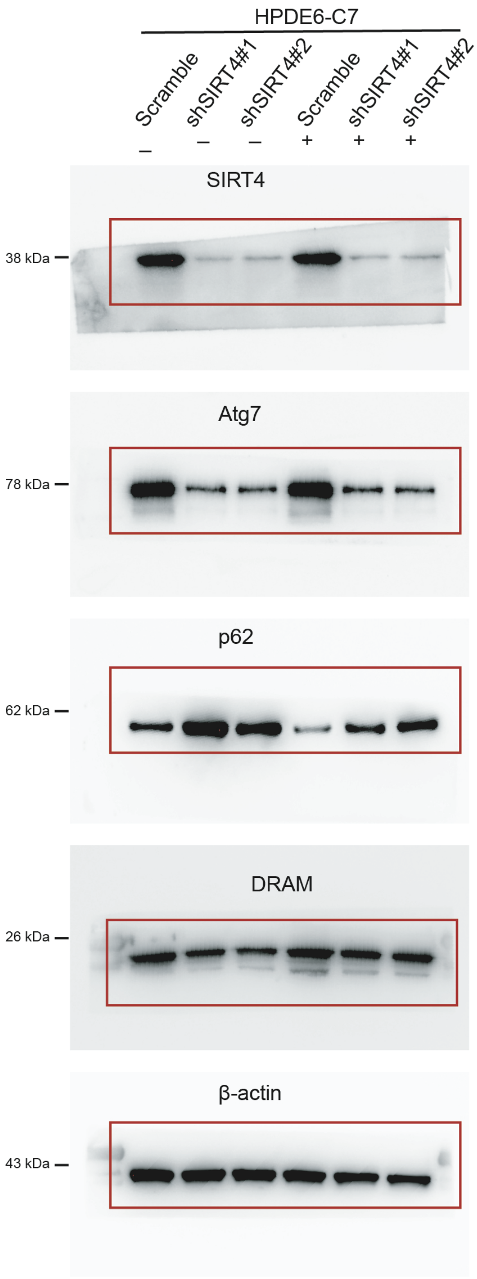

sFig 2M

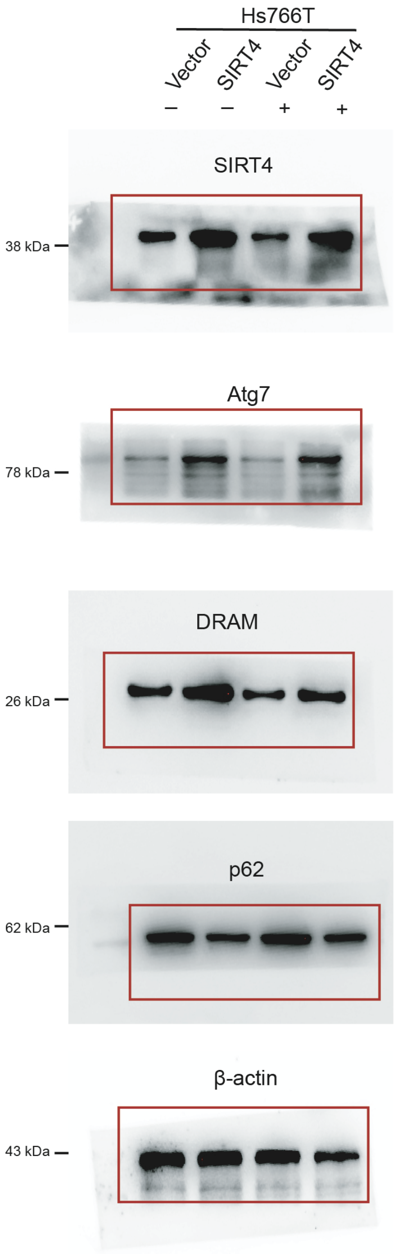

Supplement: Supplementary file 11 — Original picture of western boltting [file 41418_2022_1063_MOESM11_ESM.pdf]
